# CCBE1 promotes tumor lymphangiogenesis and is negatively regulated by TGFβ signaling in colorectal cancer

**DOI:** 10.7150/thno.39740

**Published:** 2020-01-16

**Authors:** Jinglue Song, Wei Chen, Xuewei Cui, Zhenyu Huang, Dongpeng Wen, Yili Yang, Wei Yu, Long Cui, Chen-Ying Liu

**Affiliations:** 1 Department of Colorectal and Anal Surgery, Xinhua Hospital, Shanghai Jiao Tong University School of Medicine, Shanghai 200092, China; 2 State Key Laboratory of Genetic Engineering and Collaborative Innovation Center for Genetics and Development, School of Life Sciences and Zhongshan Hospital, Fudan University, Shanghai 200438, China; 3 Suzhou Institute of Systems Medicine, Center for Systems Medicine Research, Chinese Academy of Medical Sciences, Suzhou, Jiangsu 215123, China; 4 Shanghai Colorectal Cancer Research Center, Shanghai 200092, China; 5 Department of anesthesiology, Shanghai Ninth People's Hospital, Shanghai Jiao Tong University School of Medicine, Shanghai 200011, China; 6 The First Department of Gastrointestinal Surgery, Henan Provincial People's Hospital, People's Hospital of Zhengzhou University, People's Hospital of Henan University, Zhengzhou, Henan 450003, China.

**Keywords:** CCBE1, colorectal cancer, tumor lymphangiogenesis, lymphatic metastasis, TGF-β

## Abstract

Collagen and calcium-binding EGF domain-1 (CCBE1) is essential for lymphatic vascular development as it promotes vascular endothelial growth factor C (VEGFC) proteolysis. A recent study reported that CCBE1 was overexpressed in epithelial colorectal cancer (CRC) cells; however, the role of CCBE1 in tumor lymphangiogenesis and the mechanism underlying dysregulated CCBE1 expression in CRC remain undefined.

**Methods:** The role of CCBE1 in tumor lymphangiogenesis and lymphatic metastasis was investigated using human lymphatic endothelial cells (HLECs) model *in vitro*, and a hindfoot lymphatic metastasis model *in vivo*. Immunochemistry analysis was performed to assess CCBE1 expression, prognostic value and correlation with clinicopathological characteristics in CRC. The biochemical function and transcriptional regulatory mechanism of CCBE1 were explored by western blot, qPCR, and chromatin immunoprecipitation.

**Results**: Cancer cell-derived CCBE1 enhances VEGFC proteolysis* in vitro*, facilitates tube formation and migration of HLECs *in vitro*, and promotes tumor lymphangiogenesis and lymphatic metastasis *in vivo*. In addition to CRC cells, tumor stroma within CRC tissue shows high CCBE1 expression, which is associated with high lymphatic vessel density, increased lymph node metastasis and poor prognosis. Cancer-associated fibroblasts (CAFs) express and secret CCBE1, thereby contributing to VEGFC maturation and tumor lymphangiogenesis in CRC. Transforming growth factor beta (TGF-β) downregulates the transcription and lymphangiogenic function of CCBE1 in CAFs and CRC cells through direct binding of SMADs to CCBE1 gene locus. Inactivation of the TGF-β pathway correlates with increased CCBE1 expression in CRC.

**Conclusion**: Our results demonstrate the protumorigenic role of CCBE1 in promoting lymphangiogenesis and lymphatic metastasis in CRC, revealing a new mechanism by which loss of TGF-β signaling promotes CRC metastasis.

## Introduction

Colorectal cancer (CRC) is the third most common cancer and the fourth leading cause of cancer death globally 1. The spread of tumor cells into lymphatic circulation is associated with poor prognosis and contributes to distant metastasis in CRC 2, 3. Compared with stage II (no lymphatic metastasis) CRC patients, stage III CRC patients have a significantly lower five-year survival rate (59.5% versus 82.5%) 4. Lymphatic metastases are regarded as important precursors of some distant metastases; this idea has been confirmed by the identification of common subclones in some CRC cases 3. Tumor-induced lymphangiogenesis and the remodeling of existing lymphatics are crucial for lymphatic metastasis, including in CRC 5, 6. Vascular endothelial growth factor C (VEGFC), through binding to its receptor VEGFR3 on lymphatic endothelial cells (LECs), promotes LEC proliferation and migration, resulting in hyperplasia, dilation and increased permeability of lymphatic vessels in the tumor microenvironment 5-7. High VEGFC and VEGFR3 expression has been observed in CRC, and these high levels are positively correlated with lymphangiogenesis, lymphatic metastasis and poor prognosis 8, 9.

After translation, VEGFC is secreted and then stepwise proteolytically cleaved to generate several forms with different degree of receptor binding capacity and biological activity for VEGFR3 and VEGFR2 10. Secreted VEGFC is expressed as an intact monomeric 58 kDa precursor that is first processed to a 43 kDa polypeptide, then C-terminally processed to the 29/31 kDa pro-VEGFC form and finally fully processed to the 21 kDa mature form, which has the highest activity towards VEGFR3 10, 11. Recent studies have implicated collagen and calcium-binding EGF domain-1 (CCBE1) and a disintegrin and metalloproteinase with thrombospondin motifs 3 (ADAMTS3) in regulating VEGFC proteolysis 11, 12. Studies in zebrafish and Ccbe1 KO mice have shown that CCBE1 plays an indispensable role in lymphangiogenesis through processing VEGFC during embryonic development 13-15. In humans, CCBE1 gene mutations are associated with Hennekam syndrome, a generalized lymphatic dysplasia disease with severe lymphedema 16. Although CCBE1 plays a vital role in embryonic lymphatic development, its role in tumor lymphangiogenesis and lymphatic metastasis remains unknown 5.

Intriguingly, studies reporting the roles of CCBE1 in cancer are contradictory. CCBE1 expression was first reported to be downregulated in ovarian cancer and breast cancer, and loss of CCBE1 was shown to increase tumor cell migration, implicating CCBE1 as a tumor suppressor 17, 18. Recently, CCBE1 was found to be oncogenic, conferring resistance to imatinib in GSIT by enhancing tumor angiogenesis 19. Whilst this manuscript was under preparation, a clinicopathological study reported that CCBE1 was overexpressed in epithelial CRC cells, which was associated with poor prognosis 20. However, the role of CCBE1 in tumor lymphangiogenesis during CRC progression has not been defined *in vitro* and *in vivo,* and the mechanism underlying dysregulated CCBE1 expression in CRC remains obscure. Moreover, CCBE1 expression in the tumor environment and its clinicopathological implication in CRC remain undescribed. In this study, we report that CCBE1 secreted by CRC cells enhances VEGFC processing, *in vitro* lymphangiogenesis and *in vivo* lymphatic metastasis. CCBE1 was expressed in not only CRC cells but also the tumor stroma; CCBE1 expression in each location was correlated with poor prognosis and lymph node (LN) metastasis. Transforming growth factor beta (TGF-β) signaling inhibited CCBE1 expression through the binding of SMADs to the enhancer regions of CCBE1 in CRC. VEGFC proteolytic processing and HLEC tube formation were inhibited by TGF-β and partially rescued by CCBE1 overexpression in SW837 cells and cancer-associated fibroblasts (CAFs). Our study elucidates the lymphangiogenic role of CCBE1 in CRC progression and reveals the mechanism by which TGF-β suppresses tumor lymphangiogenesis.

## Results

### CCBE1 secreted by CRC cells contributes to VEGFC proteolysis and maturation

**Figure 1:**
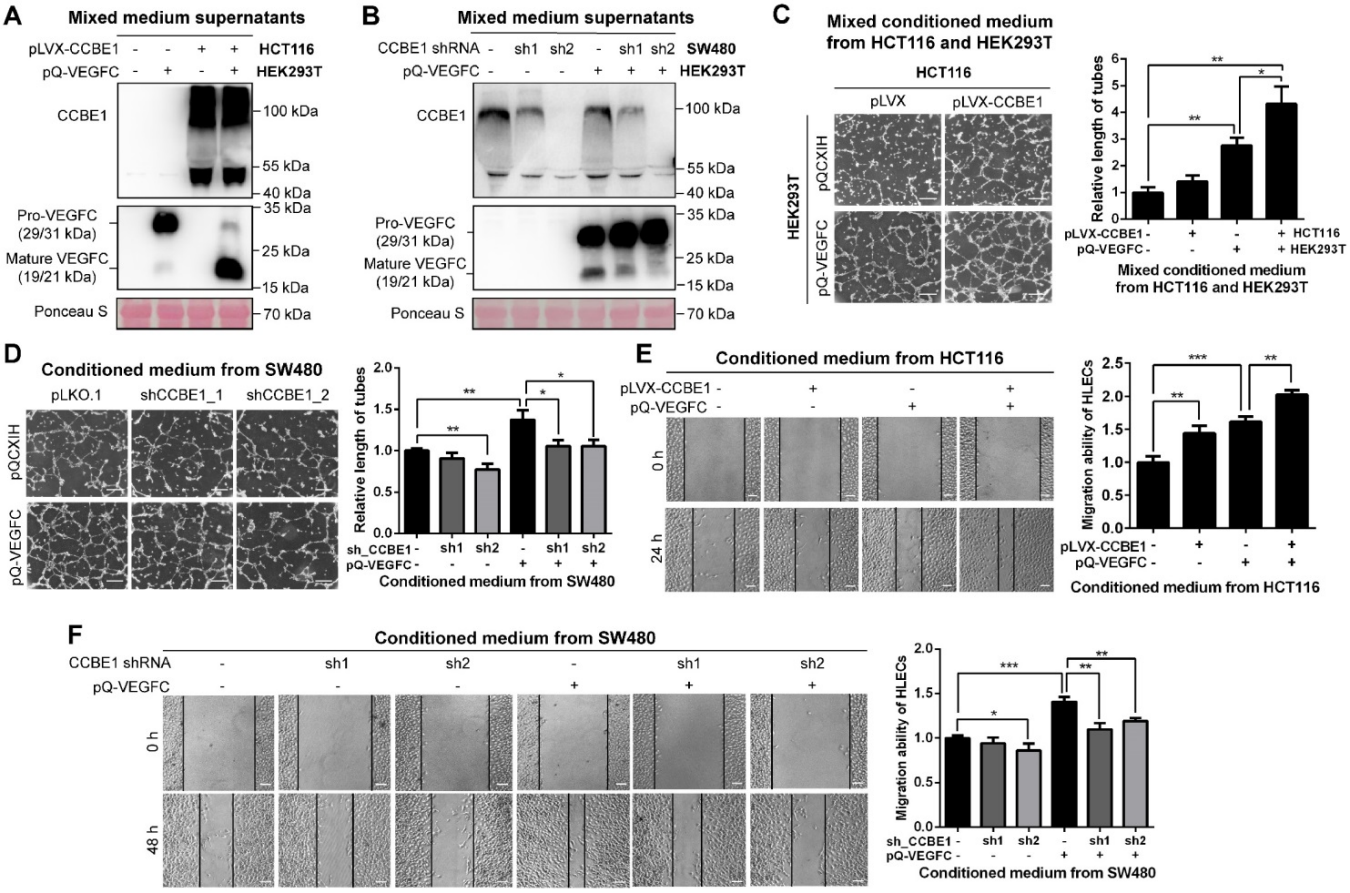
** CCBE1 secreted by CRC cells promotes VEGFC proteolysis and lymphangiogenesis *in vitro.***(A, B) Western blot analysis of CCBE1, pro-VEGFC and mature VEGFC protein levels in the indicated mixed conditioned medium. Conditioned medium from the indicated stable HCT116 (A) and SW480 (B) cells was mixed with conditioned medium from VEGFC-expressing 293T cells (1:1), incubated overnight and analyzed by western blotting. Ponceau S staining was used to control for equal loading of supernatant samples. (C, D) HLEC tube formation assay. HLECs were cultured with mixed conditioned medium from the indicated CCBE1-expressing HCT116 cells and VEGFC-expressing 293T cells (1:1) (C) or with conditioned medium from the indicated CCBE1-knockdown SW480 cells overexpressing VEGFC (D). Scale bars: 100 μm. *P<0.05, **P<0.01 by Student's t-test. (E, F) Would healing assay of HLECs cultured with conditioned medium from the indicated HCT116 (E) and SW480 cells (F). Scale bars: 20 μm. *P<0.05, **P<0.01, ***P<0.001 by Student's t-test.

Since the mRNA level of CCBE1 is lower in HCT116 cells and higher in SW480 cells (Figure S1A), and SW480 is derived from a primary adenocarcinoma of the colon with lymph node metastasis 21.To clarify the biochemical function of CCBE1 secreted by CRC cells, we established HCT116 cells with stable CCBE1 overexpression and SW480 cells with stable CCBE1 knock down by two independent shRNAs (Figure S1B). Western blot analysis of HCT116 cell lysates and culture supernatants showed that CCBE1 was expressed as an approximately 44 kDa protein in lysates and as a smear from 50 kDa to 130 kDa in the supernatant (Figure S1C), consistent with a previous report in 293T cells 11. As CCBE1 has been well studied regarding its role in VEGFC processing, we also established 293T cells stably expressing VEGFC. By mixing the supernatants of HCT116 or SW480 cells with those of 293T cells, we found that pro-VEGFC (29/31 kDa) in the supernatant was largely reduced and processed to the mature form (19/21 kDa) after CCBE1 overexpression (Figure 1A), while less mature VEGFC was detected after CCBE1 knockdown (Figure 1B), indicating that CCBE1 secreted by CRC cells participates in the processing of VEGFC secreted by other cell types. We also stably expressed VEGFC in CCBE1-overexpressing HCT116 and SW480 cells and CCBE1-knockdown SW480 cells and observed similar results: VEGFC proteolytic processing was enhanced by CCBE1 overexpression but decreased by CCBE1 knockdown (Figure S1D and S1E). These data demonstrated that CCBE1 secreted by CRC cells promotes the proteolytic processing of VEGFC produced by CRC cells or other cells in the tumor microenvironment.

### CCBE1 promotes CRC cell-induced lymphangiogenesis *in vitro*

First, we observed that overexpression or knockdown of CCBE1 did not affect the proliferation of HCT116 and SW480 CRC cells (Figure S1F). Next, to further explore the role of CCBE1 in tumor lymphangiogenesis, tube formation and wound healing assays were performed with HLECs. Conditioned medium from 293T and SW480 cells overexpressing VEGFC enhanced HLEC tube formation (Figure 1C and 1D) and migration (Figure 1E and 1F), suggesting that our system of analyzing HLEC tube formation and migration was feasible. The conditioned medium from HCT116 cells overexpressing CCBE1 only mildly promoted HLEC tube formation and migration (Figure 1C and 1E), perhaps due to the relatively low expression of endogenous VEGFC in HCT116 cells. However, a mixture of conditioned medium from CCBE1-overexpressing HCT116 cells and VEGFC-overexpressing 293T cells significantly enhanced VEGFC-induced HLEC tube formation and migration (Figure 1C and 1E). Consistently, CCBE1 knockdown in SW480 cells attenuated the pro-lymphangiogenic effect of VEGFC (Figure 1D and 1F). Taken together, our data showed that CCBE1 expression in CRC cells cooperate with VEGFC to promote lymphangiogenesis *in vitro*.

### CCBE1 promotes LN metastasis *in vivo*

**Figure 2:**
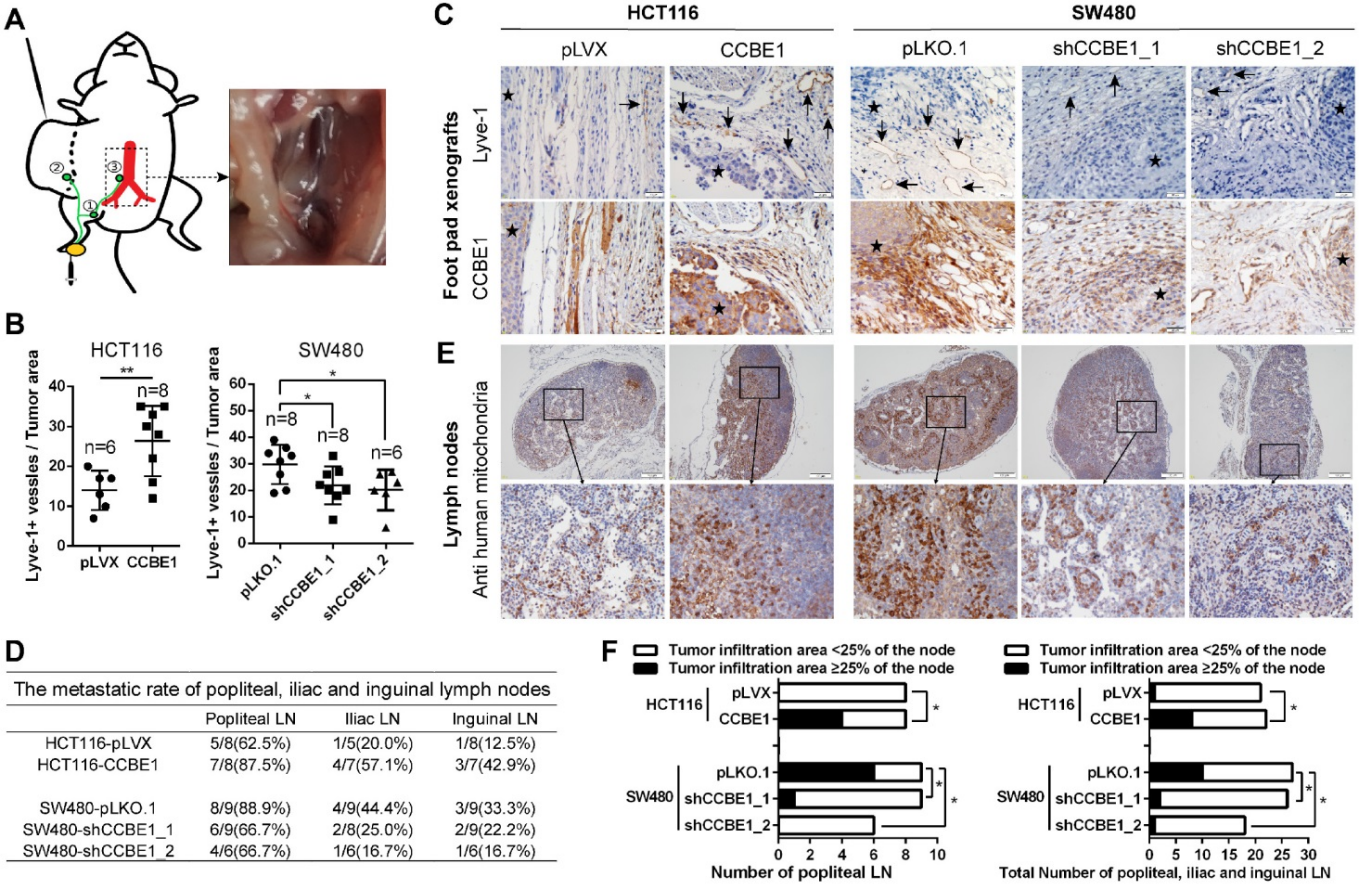
** CCBE1 promotes lymphangiogenesis and LN metastasis *in vivo*.** (A) Illustration of the hindfoot lymphatic metastasis mouse model. Yellow oval: CRC cell line-derived xenografts in the footpad. Green curves and circles: lymphatic drainage and LN. 1. Popliteal LN. 2. Inguinal LN. 3. Iliac LN. Red branches: aorta abdominalis and iliac artery. Right panel: representative image of metastasis in the right iliac LN. (B) Immunohistochemical analysis of CCBE1 and Lyve-1 (lymphatic vessel number) in CRC cell line-derived xenografts in the mouse footpad. Student's t-test was performed to assess statistical significance. *P<0.05, **P<0.01. (C) Representative images of Lyve-1(+) lymphatic vessels. CCBE1 protein expression in the same field is shown. Black arrows: Lyve-1(+) lymphatic vessels. Stars: CRC cells. Scale bars: 20 μm. (D) The metastasis ratios in dissected popliteal, iliac and inguinal LNs. (E) Representative images of metastatic LNs. Metastatic CRC cells in the LN were detected by immunohistochemistry using an anti-human mitochondria antibody. Scale bars: 100 μm. (F) Tumor infiltration in ≥25% of the node was used as a cutoff value to evaluate the degree of metastasis. Fisher's exact test was performed to assess the statistical significance of results in the sentinel popliteal LNs. A chi-square test with the continuity correction was performed to assess the statistical significance of results in the total popliteal, iliac and inguinal LNs. *P<0.05.

To validate the biological function of CCBE1 in tumor lymphangiogenesis and lymphatic metastasis *in vivo,* we established a hindfoot lymphatic drainage model 22-25. In this model, control/CCBE1-overexpressing HCT116 cells and control/CCBE1-knockdown SW480 cells implanted in mouse foot pads can invade newly generated or adjacent existing lymphatic vessels to obtain access to the hindfoot lymphatic drainage system, mainly through the sentinel popliteal LN to the iliac LN, with minor drainage to the inguinal LN 23 (Figure 2A). Among the primary tumors grown in foot pads, those overexpressing CCBE1 had more Lyve-1-positive lymphatic vessels, while those with CCBE1 knockdown had significantly fewer Lyve-1-positive lymphatic vessels (Figure 2B and C). Next, we examined the popliteal, iliac and inguinal LNs to evaluate CRC lymphatic metastasis. We detected the metastatic tumor cells in lymph nodes by immunochemistry with a human cell-specific anti-mitochondrion protein antibody, which does not react with mouse cells and is a marker for human CRC cells 26, 27. The ratio of metastasis-positive LNs at all three levels was increased by CCBE1 overexpression and decreased by CCBE1 knockdown (Figure 2D and 2E), although the difference of metastatic ratio between control group and CCBE1 OE/KD groups based on the absolute number of metastases involved LNs was not dramatic. This could be due to the use of Nude mice rather than Nod/SCID or Nod/SCID/Gamma mice which are more susceptible to tumor spread, and difficult to find the best time window to examine the LNs. Meanwhile, CCBE1-overexpressing tumors displayed a higher number of human mitochondrion protein-positive tumor cells in sentinel LNs (popliteal LN) and all three draining nodes than control tumors (Figure 2F). Conversely, knockdown of CCBE1 in SW480 cells decreased the number of human mitochondrion protein-positive tumor cells in the LNs. Taken together, these data indicated that CCBE1 promotes tumor lymphangiogenesis and lymphatic metastasis in CRC *in vivo*.

### Tumor stromal expression of CCBE1 is correlated with tumor lymphangiogenesis and LN metastasis in CRC

**Figure 3:**
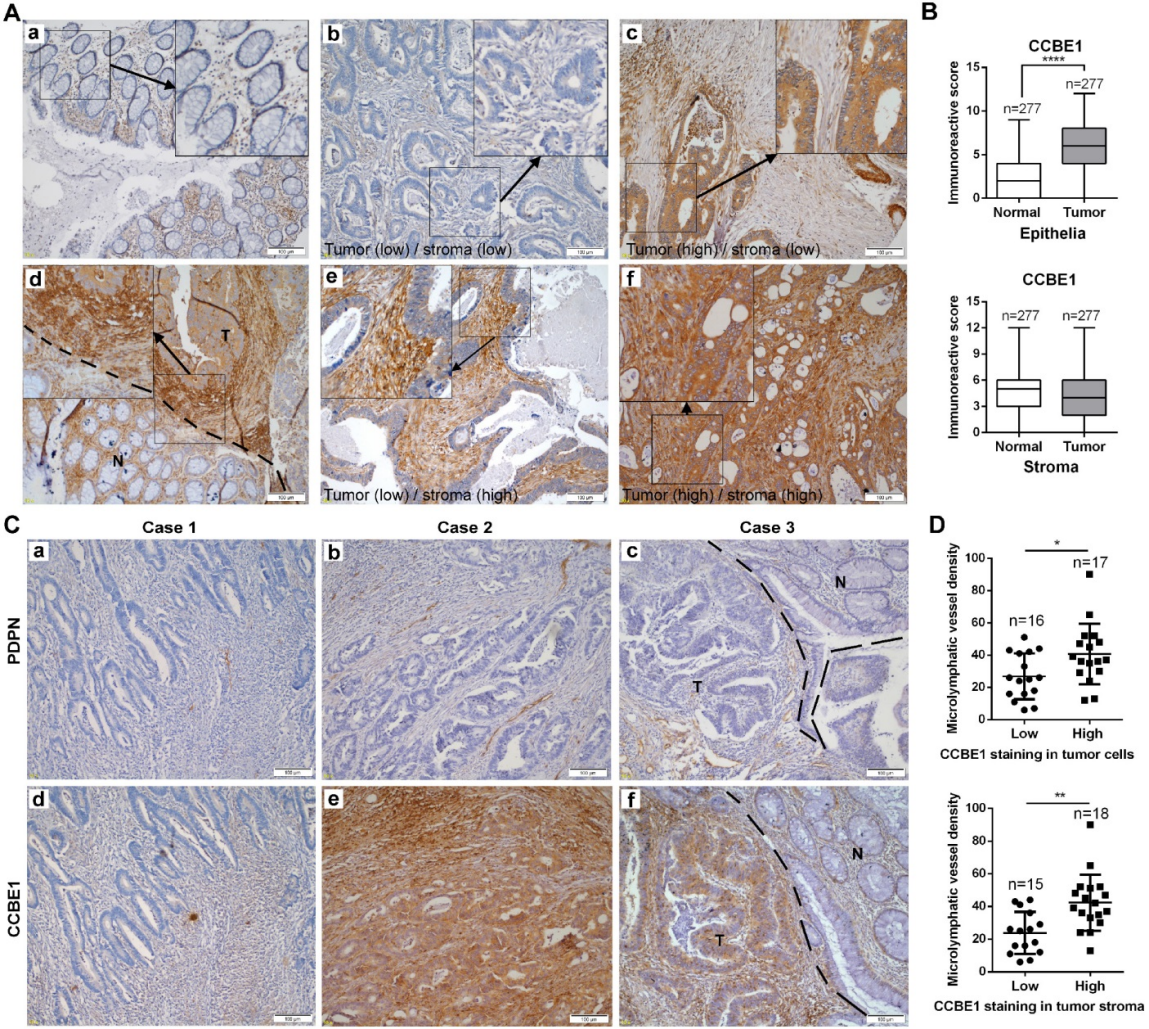
** Tumor stromal expression of CCBE1 is correlated with tumor lymphangiogenesis.** (A) Immunohistochemical analysis of CCBE1 in 277 paired CRC and normal mucosa tissues. Representative images of normal mucosa (a), low CCBE1 expression in CRC cells and stroma (b), high CCBE1 expression in CRC cells and low expression in stroma (c), CRC invasive margin (d, the boundary between tumor and normal tissue was indicated with a dashed line, T: tumor, N: normal), low CCBE1 expression in CRC cells and high expression in stroma (e), and high CCBE1 expression in both CRC cells and stroma (f). Scale bars: 100 μm. (B) Box-and-whisker plots of CCBE1 expression levels in CRC and normal epithelia (left) and stroma (right). The Mann-Whitney U test was performed to assess statistical significance. ****P<0.0001. (C, D) Immunohistochemical analysis of 33 total CRC tissue sections with CCBE1 and PDPN antibodies. (C) Representative images of the same field with low lymphatic vessel density (a) and low CCBE1 expression (d) in CRC, with high lymphatic vessel density (b) and high CCBE1 expression (e) in CRC, and with lymphatic vessel density (c) and CCBE1 expression (f) in CRC invasive margins (the boundary between tumor and normal tissue was indicated with a dashed line, T: tumor, N: normal). Scale bars: 100 μm. (D) CRC samples with high CCBE1 expression in tumor stroma or tumor cells have a high tumor lymphatic vessel density compared with those with low CCBE1 expression. PDPN (+) micro-lymphatic vessels in five high-power fields were counted and summed. Student's t-test was performed to assess statistical significance. *P<0.05, **P<0.01.

To further assess CCBE1 protein expression in CRC tissues, we also performed immunohistochemistry analysis of CRC tissue arrays consisting of 277 CRC and paired normal tissues by using a CCBE1-specific antibody from The Human Protein Atlas. Consistent with a previous report 20, CCBE1 was hardly expressed in normal epithelial cells but was significantly overexpressed in CRC cells (Figure 3A and B). Interestingly, we noticed that CCBE1 was modestly expressed in both normal and tumor stroma, with no significant differential expression between these two tissues (Figure 3A and 3B). Furthermore, similar to CCBE1 expression in tumor cells, high CCBE1 expression in tumor stroma was also correlated with advanced CRC (stage III and IV) and LN metastasis but not with other characteristics, such as pathology grade, tumor infiltration depth and distal metastasis (Table 1 and S1), indicating the potential role of tumor stromal CCBE1 expression in CRC lymphatic metastasis.

Because CCBE1 is an embryonic lymphangiogenesis factor, we next analyzed whether tumor stromal CCBE1 expression correlates with tumor lymphangiogenesis in CRC using an antibody against podoplanin (PDPN), a marker of LECs, to indicate the lymphatic vessel density in 33 CRC biopsies 28. As shown in Figure 3C, more PDPN-positive lymphatic vessels were found in CRC tissues with higher CCBE1 expression (panels a, b, d, and e). Notably, CCBE1 expression was significantly higher in tumor margins than in adjacent normal tissues (Figure 3C, panel f, and Figure 3A, panel d). Accordingly, more lymphatic vessels infiltrated tumor margins than normal tissues (Figure 3C, panel c). Statistically, CCBE1 expression in both tumor stroma and tumor cells was positively correlated with lymphatic vessel density (Figure 3D). Similar results were observed in the TCGA CRC dataset. In CRC, CCBE1 mRNA levels were remarkably correlated with the expression signatures of LYVE-1 and PDPN, two markers of LECs (Figure S1G). Taken together, these data indicated that CCBE1 in the tumor stroma might contribute to CRC lymphangiogenesis and progression.

### TGF-β suppresses the expression and lymphangiogenic function of CCBE1 in CAFs

**Figure 4:**
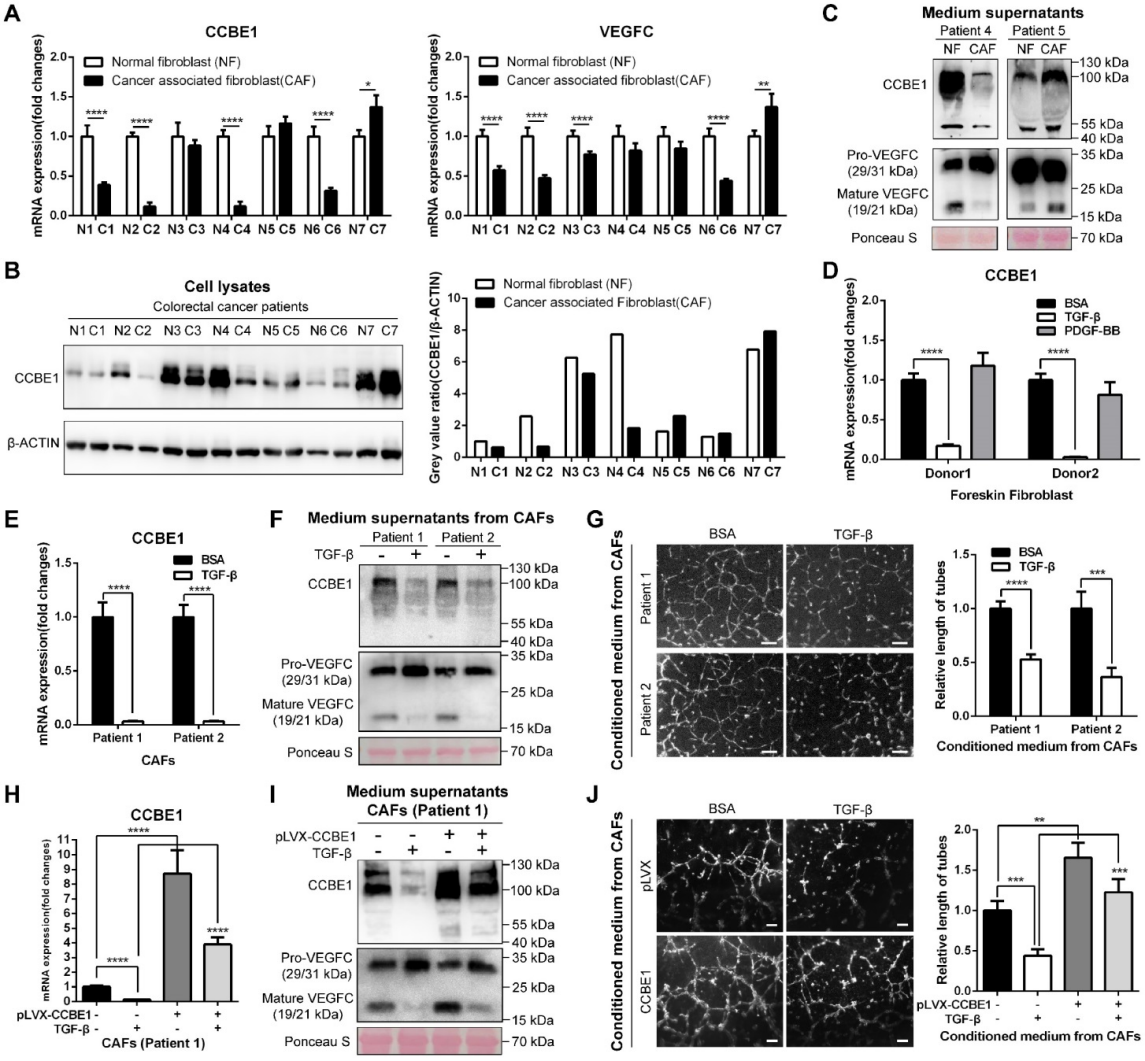
** TGF-β suppresses the expression and lymphangiogenic function of CCBE1 in CAFs.** (A) qPCR analysis of CCBE1 and VEGFC mRNA levels in paired NFs and CAFs isolated from tumor and normal mucosa tissues of CRC patients. *P<0.05, **P<0.01, ****P<0.0001 by Student's t-test. (B) Western blot of CCBE1 protein levels in lysates of 7 paired NF and CAF samples. The relative protein expression levels were quantified by grey value analysis. (C) Western blot of CCBE1, pro-VEGFC and mature VEGFC protein levels in the supernatants of 2 paired NF and CAF samples. Ponceau S staining was used to control for equal loading of supernatant samples. (D) qPCR analysis of CCBE1 mRNA expression levels in foreskin fibroblasts treated with control BSA (0.1%), TGF-β (10 ng/ml) or PDGF-BB (20 ng/ml) for 72 h to induce CAFs *in vitro*. ****P<0.0001 by Student's t-test. (E, F, G) CAFs from two CRC patients were treated with control BSA (0.1%) or TGF-β (10 ng/ml) for 72 h. (E) qPCR analysis of CCBE1 and VEGFC mRNA levels in the indicated CAFs. (F) Western blot analysis of CCBE1, pro-VEGFC and mature VEGFC protein levels in supernatants from the indicated CAFs. (G) HLEC tube formation assay with conditioned medium from the indicated CAFs. Scale bars: 100 μm. ***P<0.001, ****P<0.0001 by Student's t-test. (H, I, J) CAFs from patient 1 were treated with TGF-β and/or transfected with CCBE1 expression virus. (H) qPCR analysis of CCBE1 mRNA expression. (I) Western blot analysis of CCBE1, pro-VEGFC and mature VEGFC protein levels in supernatants of the indicated CAFs. (J) HLEC tube formation assay with conditioned medium of the indicated CAFs. Scale bars: 100 μm. **P<0.01, ***P<0.001, ****P<0.0001 by Student's t-test.

Since immunochemistry analysis of CCBE1 in CRC tissues showed strong staining in the stroma and CCBE1 mRNA levels were positively associated with the expression signatures of CAF markers, including ACAN, αSMA, CDH13, DKK3, TAGLN, and TGM2 29 (Figure S1H), we explored whether CAFs express and secrete CCBE1. First, immunofluorescent co-staining of CCBE1 and the CAF marker αSMA in CRC tissue supported that CAFs could express CCBE1 (Figure S2A). Next, we isolated and cultured 7 pairs of CAFs and primary normal fibroblasts (NFs) from fresh colorectal tissues. The isolated fibroblasts expressed the fibroblast marker αSMA but not the epithelial cell marker CK18, which was validated by an immunofluorescence assay (Figure S2B). Surprisingly, compared with paired NFs, CAFs from four patients had significantly decreased CCBE1 mRNA expression, whereas those from the other three patients showed no obvious change (Figure 4A, the left panel). CCBE1 protein levels were decreased in CAFs, consistent with the change in mRNA levels, except for the samples from patient 6 (Figure 4B). Interestingly, VEGFC mRNA levels were also decreased in CAFs in which CCBE1 mRNA levels were downregulated (patients 1, 2, 3, and 6), suggesting the decreased lymphangiogenic function of these CAFs (Figure 4A, the right panel). To explore the role of CCBE1 secreted by NFs and CAFs in VEGFC proteolysis, supernatants from patients 4 and 5 were analyzed; in these samples, VEGFC was expressed at the same level in NFs and CAFs, so decreased mature VEGFC levels could only be due to decreased CCBE1 expression, not decreased total VEGFC expression levels. Indeed, CAFs with downregulated CCBE1 mRNA levels secreted less CCBE1 than NFs, thereby compromising the proteolytic processing of VEGFC (Figure 4C). Then, we generated stable CCBE1-knockdown CAFs from two CRC patient using two independent shRNAs (Figure S2C) to further confirm the lymphangiogenic function of CCBE1 in CAFs. Consistent with its lymphangiogenic function in epithelial CRC cells, the proteolytic processing of VEGFC (Figure S2D) and HLEC tube formation (Figure S2E) were both attenuated by CCBE1-knockdown CAFs. These data indicated that CAFs express and secrete CCBE1, which can promote tumor lymphangiogenesis in CRC.

Next, we established an *in vitro-*induced CAF system by isolating foreskin fibroblasts and stimulating them with TGF-β and PDGF-BB to generate CAFs 30, 31. Intriguingly, TGF-β-induced CAFs showed dramatical downregulation of both CCBE1 and VEGFC mRNA, similar to the findings in primary CAFs compared to NFs from CRC samples (Figure 4D and S2F). PDGF-BB only weakly affected CCBE1 and VEGFC mRNA expression in cells from donor 1 (Figure 4D and S2F). We also confirmed decreased CCBE1 and VEGFC protein levels in TGF-β-induced CAFs and corresponding supernatants by western blotting (Figure S2G). In contrast to the observation in foreskin fibroblast-induced CAFs, TGF-β-treated CAFs from two patients showed weak downregulation of VEGFC mRNA expression in this primary CAF system (Figure S2H). The expression of PMEPAI and CTGF, two known TGF-β target genes, increased after TGF-β treatment, indicating the activation of TGF-β signaling in the primary CAFs (Figure S2I). Consistently, we found that TGF-β stimulation of primary CAFs dramatically inhibited CCBE1 mRNA expression (Figure 4E), VEGFC proteolytic processing (Figure 4F) and HLEC tube formation and migration (Figure 4G and S2J). Next, to further confirm that downregulation of CCBE1 mediates the suppressive function of TGF-β in lymphangiogenesis, we rescued CCBE1 expression in TGF-β-treated CAFs (Figure 4H). This rescue partially reversed the attenuated proteolytic processing of VEGFC (Figure 4I) and the inhibitory function of TGF-β on HLEC tube formation (Figure 4J). Taken together, these data indicated that CCBE1 secreted by CAFs contributes to VEGFC proteolysis and lymphangiogenesis, and these functions can be inhibited by TGF-β.

### TGF-β suppresses the expression and lymphangiogenic function of CCBE1 in CRC cells

**Figure 5:**
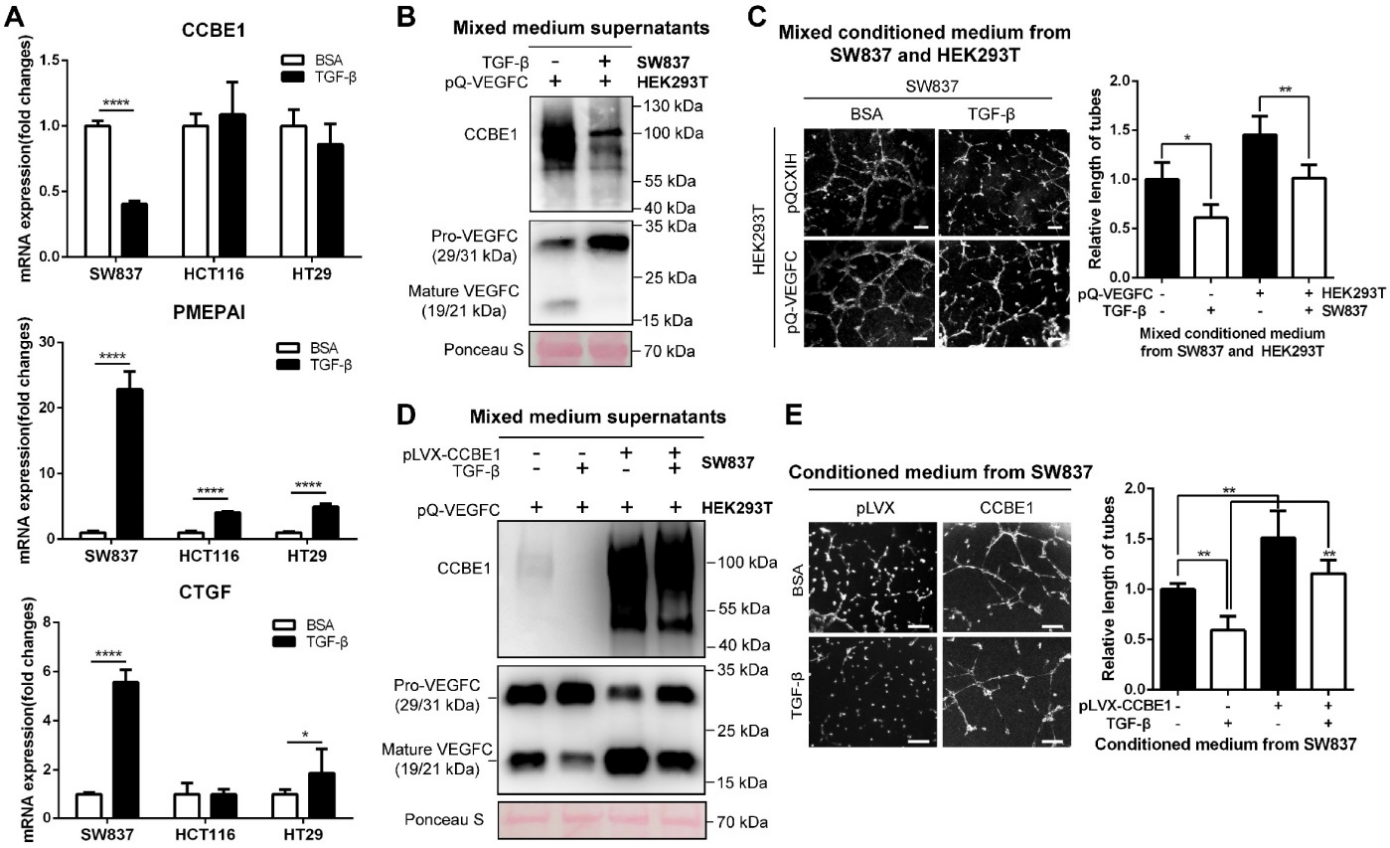
** TGF-β suppresses the expression and lymphangiogenic function of CCBE1 in CRC cells.** (A) qPCR analysis of CCBE1, PMEPAI and CTGF in a TGF-β-responsive CRC cell line (SW837) and two CRC cell lines with defective TGF-β signaling (HCT116 and HT29). Cells were treated with control BSA (0.1%) or TGF-β (10 ng/ml) for 6 h. *P<0.05, ****P<0.0001 by Student's t-test. (B, C) SW837 cells were treated with control BSA (0.1%) or TGF-β (10 ng/ml) for 48 h, and then, the supernatant was incubated overnight at 37 °C with conditioned medium from VEGFC-overexpressing 293T cells. (B) Western blot analysis of CCBE1, pro-VEGFC and mature VEGFC protein levels in the indicated supernatants. Ponceau S staining was used to control for equal loading of supernatant samples. (C) HLEC tube formation assay with the indicated conditioned medium. *P<0.05, **P<0.01 by Student's t-test. Scale bars: 100 μm. (D, E) SW837 cells were treated with TGF-β and/or transfected with CCBE1 expression virus. (D) Western blot analysis of CCBE1, pro-VEGFC and mature VEGFC protein levels in supernatants from the indicated SW837 cells. (E) HLEC tube formation assay with conditioned medium from the indicated SW837 cells. **P<0.01 by Student's t-test. Scale bars: 100 μm.

The TGF-β pathway is frequently mutated in epithelial CRC cells 32, 33. To further explore the inhibitory effect of TGF-β on CCBE1 expression in CRC, a TGF-β-responsive CRC cell line (SW837) 34 and four CRC cell lines with defective TGF-β signaling (HCT116, TGFBR2 mutant; HT29, SMAD4 mutant; SW480, SMAD4 deficient; and LoVo, SMAD2 mutant) were used to investigate the effect of TGF-β on CCBE1 gene transcription in CRC cells. The activation of TGF-β signaling was confirmed in TGF-β-treated SW837 cells by the dramatic increase in the mRNA expression of two target genes, PMEPAI and CTGF, whereas the activation of PMEPAI and CTGF expression was significantly attenuated in HCT116 and HT29 cells; these findings are consistent with those of a previous study 34 (Figure 5A). Consistent with the results in CAFs, CCBE1 mRNA was significantly downregulated by TGF-β in SW837 cells but not in HCT116, HT29, SW480 and LoVo cells (Figure 5A and Figure S3A). Although VEGFC mRNA levels were unaffected by TGF-β in SW837 cells (Figure S3B), secreted CCBE1 and VEGFC proteolysis were compromised in these cells (Figure 5B). HLEC tube formation (Figure 5C and 5E) and migration (Figure S3C) induced by SW837 cell conditioned medium were also significantly decreased after TGF-β treatment. Rescuing CCBE1 expression in SW837 cells also reversed the effects of TGF-β treatment on VEGFC proteolysis and HLEC tube formation (Figure 5D and E). These data further indicated that TGF-β inhibits the expression and lymphangiogenic function of CCBE1 in TGF-β-responsive CRC cells.

### Inactivation of the TGF-β pathway upregulates CCBE1 expression in CRC

**Figure 6:**
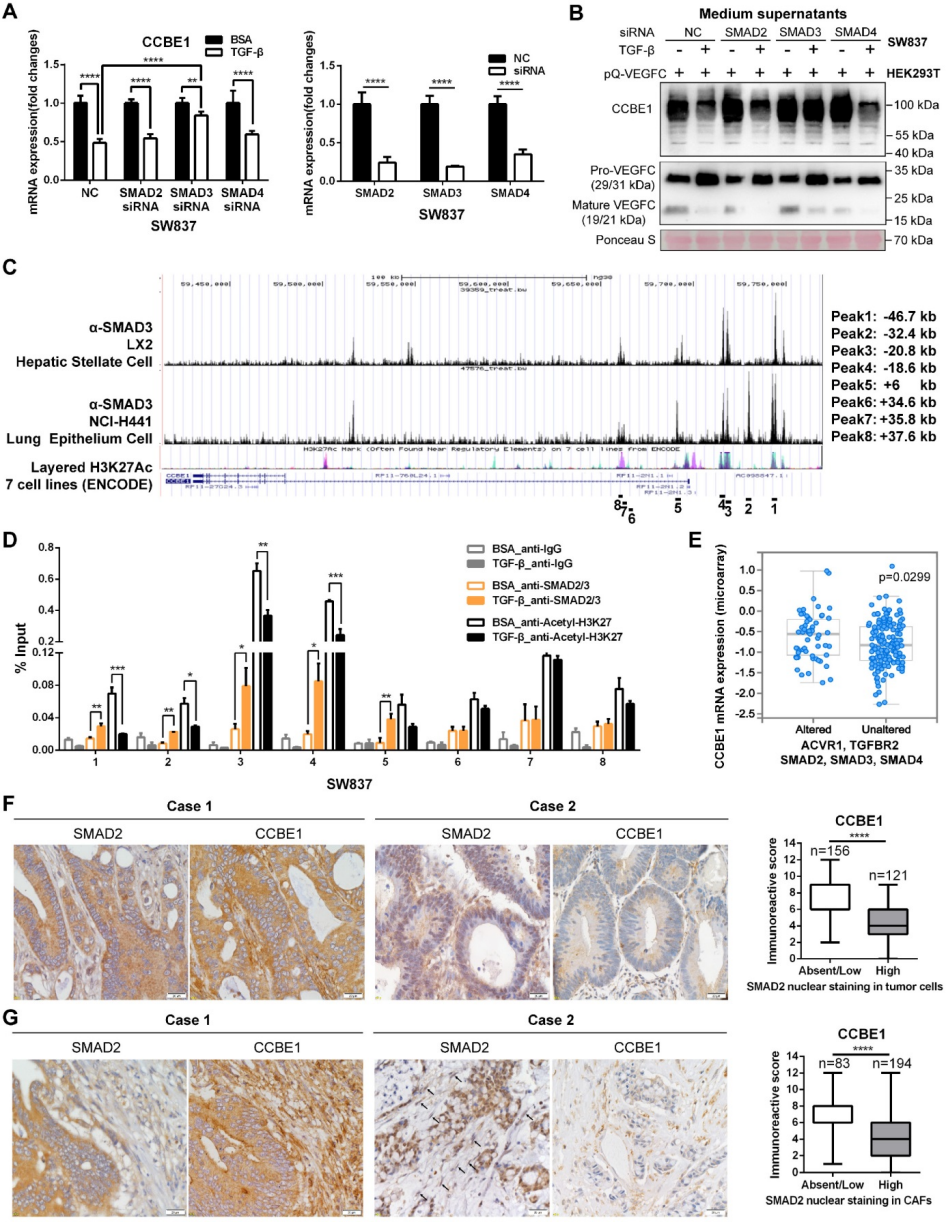
** Inactivation of the TGF-β pathway upregulates CCBE1 expression in CRC.** (A) qPCR analysis of CCBE1 and SMAD2/3/4 in SW837 cells transfected with the indicated siRNA for 72 h. Before qPCR analysis of CCBE1 mRNA levels, cells were treated with control BSA (0.1%) or TGF-β (10 ng/ml) for 6 h. **P<0.01, ****P<0.0001 by Student's t-test. (B) Western blot analysis of CCBE1 in the indicated supernatants of SW837 cells and pro-VEGFC and mature VEGFC protein levels in the indicated mixed supernatants of SW837 and HEK293T cells. Cells were treated with control BSA (0.1%) or TGF-β (10 ng/ml) for 48 h. (C) SMAD3 binds to the CCBE1 gene locus in LX2 hepatic stellate cells and NCI-H441 lung epithelial cells. ChIP-seq data for SMAD3 and H3K27Ac in 7 cell lines from ENCODE were extracted from the Cistrome database. The regions targeted by ChIP-qPCR primers are indicated. (D) ChIP analysis of SMAD2/3 binding to the CCBE1 gene locus in SW837 cells. Rabbit IgG was used as a negative control. Anti-H3K27Ac antibody was utilized to further assess the transcriptional activity of the CCBE1 gene. *P<0.05, **P<0.01, ***P<0.001 by Student's t-test. (E) Box-and-whisker plots of CCBE1 mRNA levels in the TCGA CRC dataset. Data were stratified by genetic alterations in the TGF-β-SMAD signaling pathway. The data were extracted from the cBioPortal database. (F, G) Immunohistochemical analysis of CCBE1 and nuclear SMAD2 expression in tumor cells (F) and tumor stroma (G) from 277 CRC patients. Scale bars: 20 μm. The Mann-Whitney U test was performed to assess statistical significance. ****P<0.0001.

SMAD proteins are the main downstream effectors of TGF-β, and they regulate the transcription of target genes. Thus, SMAD2/3/4 were individually knocked down to ascertain whether SMAD proteins are involved in the inhibition of CCBE1 expression by TGF-β (Figure 6A). We found that SMAD3 knockdown partially reversed the inhibitory effect of TGF-β on CCBE1 mRNA and protein expression and VEGFC proteolysis in SW837 cells (Figure 6A and B, Figure S3D). Similar results were found in CAFs (Figure S2K and L). Published chromatin immunoprecipitation (ChIP)-seq datasets showed direct binding of SMAD3 at the CCBE1 gene locus in the hepatic stellate cell line LX2 and the lung epithelial cell line NCI-H441 35. Interestingly, the SMAD3 binding peaks overlapped with the active gene transcription mark H3K27Ac in the ENCODE database (Figure 6C). To further explore whether SMAD3 directly inhibits CCBE1 gene transcription, we performed ChIP-qPCR analysis using ChIP-grade SMAD2/3 and H3K27Ac antibodies in SW837 cells. We found that SMAD2/3 were recruited to the enhancer region 1 (SMAD3 ChIP-sequence Peak 1, -46.7 kb), region 2 (SMAD3 ChIP-sequence Peak 2, -32.4 kb), region 3 (SMAD3 ChIP-sequence Peak 3, -20.8 kb) and region 4 (SMAD3 ChIP-sequence Peak 4, -18.6 kb) of the CCBE1 gene after TGF-β treatment in SW837 cells (Figure 6D). Meanwhile, H3K27Ac levels in these enhancer regions were significantly reduced after TGF-β treatment, consistent with the downregulation of CCBE1 mRNA expression, indicating that TGF-β downregulated CCBE1 expression through the activation of SMADs, which directly bound and inhibited CCBE1 gene transcription in CRC cells (Figure 6D). To further explore if the binding of SMAD2/3 to these four enhancer regions modulates CCBE1 expression, we cloned these enhancer regions into the pGL3-promoter plasmids and assessed the relative luciferase activity of these enhancer regions. The luciferase assay showed that TGF-β treatment decreased the relative luciferase activity of the enhancer region 3 (-20.8 kb) and region 4 (-18.6 kb), indicating that the region 3 (-20.8 kb) and region 4 (-18.6 kb) are the functional binding regions of SMAD2/3 response to the TGF-β (Figure S3E).

Defects in the TGF-β pathway are common in CRC. Next, we explored whether CCBE1 expression correlates with inactivation of the TGF-β pathway. First, analysis of the TCGA CRC dataset showed that CCBE1 mRNA levels were significantly higher in CRC samples with alterations in the TGF-β signaling pathway (Figure 6E). SMAD2 nuclear staining by immunohistochemistry is considered a marker of TGF-β activation. Nuclear SMAD2 expression was found in tumor cells of 45% of CRC samples (Figure 6F) and in tumor stroma of 70% of cases (Figure 6G), in which CCBE1 expression was significantly lower than in the samples with cytoplasmic SMAD2 staining. Taken together, our data demonstrated that TGF-β inhibits CCBE1 gene transcription through activation and direct binding of SMADs in CRC cells and CAFs. Defects in the TGF-β pathway in CRC mitigate the inhibitory effects of TGF-β, resulting in high CCBE1 expression, enhanced lymphangiogenesis and poor prognosis.

### Tumor stromal expression of CCBE1 is a poor prognostic marker for CRC

**Figure 7:**
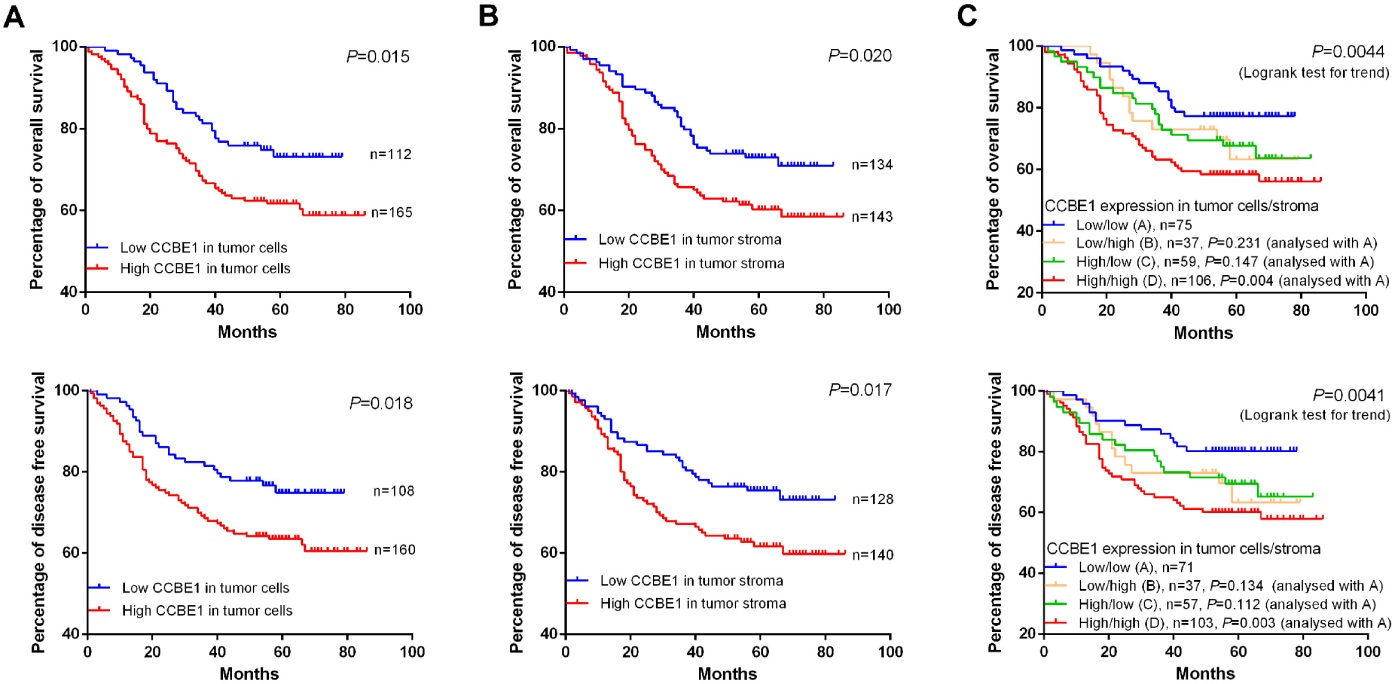
** CCBE1 expression is a poor prognostic marker for CRC.** Kaplan-Meier plots of the OS and DFS of CRC patients stratified by CCBE1 protein level in CRC cells (A), tumor stroma (B) and both CRC cells and stroma (C). The log-rank test was performed to assess statistical significance.

Consistent with the findings of a recent study, we confirmed that CCBE1 was overexpressed in epithelial CRC cells, and this overexpression was associated with poor prognosis (Figure 7A). Next, we explored the prognostic value of tumor stromal expression of CCBE1 in our cohort of CRC samples. Interestingly, although CCBE1 was not differentially expressed between normal and tumor stroma tissues, high CCBE1 protein expression in tumor stroma was associated with shorter overall survival (OS) and disease-free survival (DFS) in CRC (Figure 7B). Approximately 38.3% of CRC samples had high CCBE1 expression in both tumor cells and stroma, and these patients had the worst OS and DFS (Figure 7C). Moreover, univariate and multivariate analyses showed that CCBE1 expression in tumor cells (Table S2), tumor stroma (Table S3) or both tumor cells and stroma (Table 2) was an independent prognostic factor in CRC. Our results indicated that tumor stromal expression of CCBE1 is associated with poor prognosis in CRC and that high CCBE1 expression in both epithelial CRC cells and tumor stroma is a more accurate poor prognostic marker for CRC.

## Discussion

Lymphatic metastasis is a poor prognostic indicator for CRC. Multiple studies have demonstrated the essential role of tumor lymphangiogenesis in lymphatic metastasis in multiple cancers, including CRC 6, 8, 36-38. The VEGFC-VEGFR3 and VEGFD-VEGFR3 axes are considered the major drivers of tumor lymphangiogenesis 5. Both VEGFC and VEGFD are activated by proteolytic processing. Although these proteins bind to the same receptor, their proteolytic processing is different. The secreted protein CCBE1 interacts with and activates the metalloproteinase ADAMTS3 to cleave VEGFC but not VEGFD 12. Cleaved VEGFC is then able to activate VEGFR3 with enhanced potency. The indispensable role of CCBE1 in embryonic lymphangiogenesis has been well studied in zebrafish and Ccbe1 KO mice 11, 14, 15, but the roles of CCBE1 in tumor lymphangiogenesis and lymphatic metastasis remain unknown 5. In this study, we clearly show that overexpression of CCBE1 in CRC cells promotes VEGFC proteolysis and activation and that CCBE1 enhances tumor lymphangiogenesis and lymphatic metastasis in CRC. In addition, we show that CCBE1 is also expressed by CAFs, thereby promoting VEGFC proteolysis and lymphangiogenesis *in vitro*, although the *in vivo* lymphangiogenic function of CAF-derived CCBE1 should be explored in future studies. Both CCBE1 and VEGFC can be co-expressed in the CRC cells and CAFs. It has been reported that co-transfection of VEGFC with CCBE1 facilitates the release of VEGFC 11. Thus, besides activating VEGFC processing, overexpression of CCBE1 in CRC could also enhance the release of VEGFC to promote tumor lymphangiogenesis. Numerous studies have reported that multiple factors affect lymphatic metastasis by modulating VEGFC expression 25, 39. Based on the role of CCBE1 in VEGFC activation, it is rational that CCBE1 plays an oncogenic role, enhancing tumor lymphangiogenesis and lymphatic metastasis.

CCBE1 was first reported as a tumor suppressor, and *CCBE1* mRNA levels were shown to be decreased in ovarian and breast cancer 17, 18. Similar to the downregulation of CCBE1 mRNA levels in these two types of cancer, TCGA CRC data showed significantly decreased CCBE1 mRNA expression in CRC (Figure S3F). However, CCBE1 expression was undetectable in normal colonic cell lines but was high in CRC cell lines (Figure S1A). Our studies demonstrate that CCBE1 protein is expressed in tumor cells and stroma but hardly in normal colonic epithelial cells, which indicates that CCBE1 is overexpressed in CRC cells. The downregulation of CCBE1 mRNA in CRC tissues could be due to the mixture of cell types within a tumor, particularly the presence of some CAFs with decreased CCBE1 mRNA levels. Overexpression of CCBE1 in CRC cells further demonstrated the oncogenic function of this protein, at least in CRC. In addition, transcriptional activation of CCBE1 is the primary determinant for overexpression in CRC cells, since CCBE1 mRNA expression is undetectable in normal colonic epithelial cells but significantly increased in CRC cells. In this study, we reveal a novel mechanism by which TGF-β inhibits CCBE1 transcription in both colonic epithelial cells and fibroblasts. However, how CCBE1 transcription is activated and which transcription factors enhance CCBE1 transcription in CRC cells should be explored in the future.

TGF-β can function as an oncogene or a tumor suppressor in a cell context-dependent manner 40. CRCs normally harbor genetic alterations of TGF-β pathway components. TGFBR2, SMAD2, SMAD3 and SMAD4 mutations are present in approximately 30% of CRC cases 41. Thus, TGF-β plays a tumor suppressive role in colonic epithelial cells. However, TGF-β expression is a poor prognostic marker for CRC. Previous reports revealed that TGF-β can promote CRC cell survival and metastasis by activating CAFs to induce and secrete IL-11, which acts on CRC cells 42. Indeed, studies have found that all poor prognostic CRC subtypes share a gene expression pattern correlated with TGF-β-stimulated tumor stromal cells 43, 44. In contrast to the pro-metastatic effect of TGF-β, its first reported role was as an inhibitor of lymphangiogenesis in cancer 45. TGF-β signaling in LECs inhibits proliferation and migration, and inhibition of TGF-β signaling promoted lymphangiogenesis in a pancreatic cancer xenograft model 45. On the other hand, TGF-β was also reported to promote lymphangiogenesis by increasing VEGFC expression in some contexts, such as in cervical cancer cells 39, mesothelial cells 46, renal tubular epithelial cells and macrophages 47. The role of TGF-β in lymphangiogenesis is also dependent on cell context. Our studies show that TGF-β inhibits CCBE1 expression in both CRC cells and CAFs, resulting in less VEGFC proteolysis and activation. These data indicate that TGF-β plays a suppressive role in tumor lymphangiogenesis, probably through inhibiting CCBE1 expression and function in CRC. Consistently, a lack of SMAD4 expression 8 and SMAD4 loss by chromosome 18q deletion 48 were reported to be positively correlated with lymphatic vessel count and lymphatic metastasis in CRC. Our studies also reveal that TGF-β inactivation and genetic alterations in the TGF-β pathway are positively associated with CCBE1 expression in CRC. Thus, TGF-β inactivation in CRC cells could allow tumor cells to overcome the inhibitory effect of TGF-β on tumor lymphangiogenesis.

Multiple studies have reported that the factors involved in lymphangiogenesis and lymphatic remodeling are correlated with cancer patient outcomes 5. In CRC, VEGFC expression is a poor prognostic marker for DFS and OS, and VEGFC is an independent risk factor for LN metastasis 8. Reasonably, as the regulator of VEGFC proteolysis and activation, CCBE1 in epithelial CRC cells was reported in both a recent study and our study to be prognostic for DFS and OS. In addition, our study further showed that high CCBE1 expression in tumor stroma is an independent poor prognostic marker for DFS and OS in CRC. Thus, high CCBE1 expression in both epithelial cells and tumor stroma is a more accurate prognostic marker for CRC. Overall, this study demonstrates the protumorigenic role of the embryonic lymphangiogenic factor CCBE1 in lymphatic metastasis in CRC and reveals a novel mechanism by which TGF-β inhibits lymphangiogenesis through inhibiting CCBE1 expression.

## Methods

### Clinical sample collection

All clinical sample collection was approved by Xinhua Hospital Ethics Committee, Affiliated with Shanghai Jiaotong University School of Medicine. Informed consent was obtained for the use of all clinical samples. All human CRC and normal tissues were collected in the Department of Colorectal and Anal Surgery, XinHua Hospital, Shanghai Jiao Tong University School of Medicine, from January 2008 to December 2016. Foreskin tissues were collected from 2 donors in the Department of Urinary Surgery. A total of 277 paired CRC and normal colorectal tissues were used to prepare tissue arrays. Another 7 paired fresh samples were collected for the isolation of primary CAFs and NFs. Another 33 CRC samples were collected and sectioned for the immunohistochemical analysis of lymphatic vessels and CCBE1 expression.

### Immunohistochemistry

Experiments were performed as previously described 49. CCBE1 staining in tumor and normal tissues was scored according to the following standards: staining intensity was classified as 0 (no staining), 1 (weak staining), 2 (moderate staining) or 3 (strong staining); and the percentage of staining was designated as 1 (<25%), 2 (25-50%), 3 (51-75%) or 4 (>75%). For each section, the semi-quantitative score was calculated by multiplying these two values (total score ranged from 0 to 12). Two histopathologists were reviewed the slides and scored the staining in a blinded manner. The stroma was defined as the region surrounding normal or tumor epithelial cells in the evaluated field, including fibroblasts and other stromal cells, extracellular matrix but not identifiable blood/lymphatic vessels or muscular tissue.

### Cell culture and reagents

HEK293T, HCT116, SW480, SW837, HT29, LoVo, CCD 841, and NCM460 cells were cultured in DMEM/High Glucose (HyClone) supplemented with 10% fetal bovine serum (Gibco), 100 units/ml penicillin and 100 units/ml streptomycin. HLECs were cultured in modified RPMI medium (HyClone) supplemented with 10% fetal bovine serum (Gibco), 100 units/ml penicillin and 100 µg/ml streptomycin. Foreskin fibroblasts, primary NFs and CAFs were isolated refer to described previously 30 cultured in complete Fibroblast Growth Medium-2 (Lonza). The protocol was elaborated below. The following cell culture reagents and antibodies were used: recombinant human TGF-β1 (PeproTech), recombinant human PDGF-BB (PeproTech), anti-CCBE1 antibody (Atlas Antibodies, HPA041374), anti-VEGFC antibody (E-6, Santa Cruz Biotechnology), anti-SMAD2 antibody (D43B4, CST), anti-SMAD2/3 antibody (D7G7, CST), anti-acetyl-Histone H3 (Lys27) antibody (D5E4, CST), normal rabbit IgG (CST), anti-alpha smooth muscle actin (α-SMA) antibody (Abcam), anti-Cytokeratin 18 antibody (Abcam), anti-human D2-40 (PDPN) antibody (Dako), anti-mouse Lyve-1 antibody (ALY7, eBioscience) and anti-human mitochondria antibody (Abcam).

### Isolation and culture of human primary colorectal normal and cancer-associated fibroblasts

Human colorectal cancer-associated and normal fibroblasts were obtained from the resected fresh surgical cancer tissues and paired normal mucosa specimen (at least 1 cm far from the cancer margin), respectively. Tissues (average 1 cm size) were quickly washed twice with 10ml phosphate-buffered saline (PBS), and then gently washed for 20 min in 37 °C shaker with PBS containing high concentration penicillin (500 units/ml) /streptomycin (500 units/ml) and 2.5 μg/ml amphotericin-B (Hyclone). Then, the tissues were cut into pieces and digested in 10ml DMEM/High Glucose medium with collagenase IV (1mg/ml), hyaluronidase (25μg/ml), DNase (10μg/ ml) and 100 units/ml penicillin and 100 units/ml streptomycin in 37 °C shaker for 30 min. After digestion, the tissues were filtered with a 70 μm nylon cell strainer and the filtrate was centrifuged at 1000g for 10min. The cells from the pellet were cultured with Fibroblast Growth Medium-2 (Lonza) for overnight. On the next day, the suspending cells were washed away, and the attached cells were fibroblasts which were verified by CK18 and α-SMA staining. Fibroblasts were cultured in Fibroblast Growth Medium-2 (Lonza) to facilitate fibroblast growth according to the manufacture's instruction.

### Transfection, vector construction and virus production

Cells were transfected with siRNA using Lipofectamine RNAiMAX according to the manufacturer's protocol. Endogenous CCBE1 was knocked down by cloning two shRNA oligonucleotides into the pLKO.1 (puro) vector. Cell lines stably expressing CCBE1 shRNA were generated by infection of lentivirus, which was produced by HEK293T cells using envelope plasmid pMD2.G and packaging plasmid psPAX2. Human CCBE1 cDNA was cloned into the pLVX (puro) vector. Cell lines stably expressing CCBE1 cDNA were generated by infection of lentivirus, which was produced by HEK293T cells using envelope plasmid pMD2.G and packaging plasmid psPAX2. Human VEGFC cDNA was cloned into the pQCXIH (hygro) vector. Cell lines stably expressing VEGFC cDNA were generated by infection of retrovirus, which was produced by HEK293T cells using plasmid VSV.G and gag. Briefly, the cells were infected with the indicated virus for 24h. Then, the virus was removed and the cells were selected with puromycin (for pLKO.1 and pLVX) and hygromycin (for pQCXIH) for 1 week before functional experiments. The siRNA and shRNA sequences are provided in Table S4.

### Western blotting, qPCR, ChIP and Luciferase assay

All experiments were performed as previously described 49. To detect secreted CCBE1 and VEGFC in the supernatant, cells were seeded into a six-well plate with serum-free medium (5×10^6^/well). After 24 h, the cell culture medium was collected and boiled in 1X SDS loading buffer for western blotting. For the VEGFC proteolysis assay, culture medium from CCBE1-expressing CRC cells and VEGFC-expressing 293T cells was collected and mixed at a ratio of 1:1. After overnight incubation at 37 °C, the mixed culture medium was analyzed by western blotting to assess the extracellular proteolysis of VEGFC. The qPCR, ChIP primers and the primers for construction of the luciferase reporters were described in Table S4.

### HLEC tube formation assay

Growth Factor-Reduced Matrigel (50 μL, BD Biosciences) was pipetted into each well of a pre-cooled (4°C) 96-well plate and allowed to polymerize for 30 m at 37 °C. HLECs (1×10^4^) in 100 μL of conditioned medium were added to each well (3 replicates) and incubated at 37 °C and 5% CO_2_. Tube formation ability was quantified by measuring the total length of the cord and tubule structures.

### Wound healing assay

HLECs in complete medium were seeded into a six-well plate (1 × 10^6^/well). After 8 h, the medium was replaced with serum-free medium, and the cells were cultured overnight. A scratch in the cell monolayer was made with a 10 µl pipette tip, and then, the medium was replaced with serum-free conditioned medium from the indicated cells. Images were taken immediately after scratching and at the indicated time later. HLEC migration ability was quantified by measuring the blank area without cells.

### Hindfoot lymphatic drainage model

All mouse procedures were approved by Xinhua Hospital Ethics Committee, affiliated with Shanghai Jiaotong University School of Medicine. Nude mice (4-5 weeks old, male) were used. A total of 1×10^6^ cells in 40 µl of PBS were injected into the foot pads of nude mice. Mice injected with stable HCT116 and SW480 cells were sacrificed on days 21 and 28, respectively. Primary tumors in the foot pads and LNs at three positions of hindfoot lymphatic drainage (popliteal LN, iliac LN and inguinal LN) were sectioned and stained. Lyve-1-positive vessels were counted in each section. Metastatic tumor cells were indicated using an anti-human mitochondria antibody, and the LN metastasis ratio was calculated.

### Statistics

Spearman's rank-order correlation coefficient, the Kruskal-Wallis test and the Mann-Whitney U test were performed to evaluate the correlation between clinicopathological parameters and CCBE1 expression. The Kaplan-Meier method was used to estimate OS and DFS. Prognostic value was evaluated by univariate and multivariate Cox regression analyses. For each comparison, we used a Bonferroni-adjusted alpha level to determine statistical significance. All P-values correspond to two-sided tests, and P<0.05 was considered to indicate statistical significance.

## Supplementary Material

Supplementary figures and tables.

## Figures and Tables

**Table 1:** Correlation of CCBE1 expression in stroma with CRC patients' pathological and clinical features

Variables	CCBE1 expression in stroma			P-Values
	All cases (n=277)	Low (n=134)	High (n=143)	
**Age (year)**				0.406^b^
≤68	140	70(50.0%)	70(50.0%)	
>68	137	64(46.7%)	73(53.3%)	
**Gender**				0.788^b^
Male	153	74(48.3%)	79(51.7%)	
Female	124	60(48.4%)	64(51.6%)	
**Tumor site^a^**				0.476^c^
Proximal colon	66	31(47.0%)	35(53.0%)	
Distal colon	90	46(51.1%)	44(48.9%)	
Rectum	121	57(47.1%)	64(52.9%)	
**TNM staging**				
I	40	20(50.0%)	20(50.0%)	0.146^d^
II	112	62(55.4%)	50(44.6%)	
III	100	41(41.0%)	59(59.0%)	
IV	25	11(44.0%)	14(56.0%)	
Early stage(I/II)	152	82(53.9%)	70(46.1%)	**0.041^b^**
Late stage(III/IV)	125	52(41.6%)	73(58.4%)	
**Pathology grade**				0.807^d^
Well differentiated	93	44(47.3%)	49(52.7%)	
Moderately differentiated	130	67(51.5%)	63(48.5%)	
Poorly differentiated	54	23(42.6%)	31(57.4%)	
**Tumor infiltration depth**				0.525^b^
Limited under the serosa (T1/2/3)	147	72(49.0%)	75(51.0%)	
Penetrating the serosa (T4)	130	62(47.7%)	68(52.3%)	
**Regional lymph node metastasis**				**0.035^b^**
N0	161	86(53.4%)	75(46.6%)	
N1/N2	116	48(41.8%)	68(58.2%)	
**Distal metastasis**				0.979^b^
M0	252	123(48.8%)	129(51.2%)	
M1	25	11(44.0%)	14(56.0%)	
**CEA level^e^**				0.88^b^
0-10 ng/ml	203	99(48.8%)	104(51.2%)	
>10 ng/ml	68	34(50.0%)	34(50.0%)	

Abbreviations: TNM, tumor - node - metastasis; CEA, carcinoembryonic antigen. ^a^Proximal colon tumors are those arising in the cecum, ascending colon, hepatic flexure or transverse colon; distal colon tumors are those arising in the splenic flexure, descending colon or sigmoid colon. ^b^Mann-Whitney U Test. ^c^Kruskal-Wallis. ^d^Spearman. ^e^Six patients did not have CEA level tested. The bold values indicate statistically significant (P<0.05).

**Table 2:** Univariate and multivariate analysis of overall survival and disease-free survival

Variables	OS					DFS			
	Univariate HR (95%CI)	P value	Multivariate HR (95%CI)	P value		Univariate HR (95%CI)	P value	Multivariate HR (95%CI)	P value
**Tumor infiltration depth**									
Limited under the serosa (T1/2/3)	1		1			1		1	
Penetrating the serosa (T4)	1.708 (1.134-2.572)	0.01	1.332 (0.863-2.055)	0.403		1.703 (1.113-2.607)	0.014	1.309 (0.735-2.330)	0.36
**Clinical stage**									
Early stage(I/II)	1		1			1		1	
Late stage(III/IV)	2.295 (1.514-3.481)	<0.001	1.833 (1.188-2.829)	**0.006**		2.263 (1.468-3.489)	<0.001	2.277 (1.306-3.968)	**0.004**
**Pathology grade**		0.028		**0.047**			0.034		**0.049**
Well differentiated	1		1			1		1	
Moderately differentiated	1.453 (0.885-2.386)	0.139	1.405 (0.847-2.329)	**0.187**		1.505 (0.896-2.527)	0.122	2.091 (1.048-4.171)	**0.036**
Poorly differentiated	2.146 (1.225-3.759)	0.008	2.062 (1.161-3.662)	**0.014**		2.174 (1.211-3.904)	0.009	3.129 (1.460-6.705)	**0.003**
**CEA level**									
0-10 ng/ml	1		1			1		1	
>10 ng/ml	2.693 (1.775-4.085)	<0.001	2.318 (1.494-3.595)	**<0.001**		2.453 (1.572-3.825)	<0.001	2.212 (1.220-4.009)	**0.009**
**CCBE1 expression**		0.039		**0.028**			0.041		**0.048**
Both low in tumor cells and stroma	1		1			1		1	
Low in tumor cells and high in stroma	1.531 (0.731-3.206)	0.259	1.332 (0.618-2.870)	0.465		1.770 (0.819-3.828)	0.147	1.401 (0.627-3.129)	0.411
High in tumor cells and low in stroma	1.597 (0.837-3.049)	0.156	1.438 (0.736-2.808)	0.288		1.741 (0.866-3.502)	0.12	1.474 (0.713-3.045)	0.295
Both high in tumor cells and stroma	2.235 (1.279-3.906)	0.005	2.258 (1.270-4.018)	**0.006**		2.404 (1.313-4.404)	0.004	2.278 (1.219-4.258)	**0.01**

CCBE1 in tumor cells and stroma are both included.

## Abbreviations

CCBE1: Collagen and calcium-binding EGF domain-1
VEGFC: Vascular endothelial growth factor C
CRC: Colorectal cancer
HLEC: Human lymphatic endothelial cell
CAF: Cancer-associated fibroblast
NF: Normal fibroblast
TGF-β: Transforming growth factor beta
LEC: Lymphatic endothelial cell
ADAMTS3: A disintegrin and metalloproteinase with thrombospondin motifs 3
LN: Lymph node
PDPN: Podoplanin
