# Supplementary material for: CCBE1 promotes tumor lymphangiogenesis and is negatively regulated by TGFβ signaling in colorectal cancer
Source: Theranostics. 2020 Jan 16;10(5):2327–41. doi: 10.7150/thno.39740 (PMC7019157; doi:10.7150/thno.39740)
Supplement: Supplementary file 1 — Supplementary figures and tables. [file thnov10p2327s1.pdf]

Figure S1

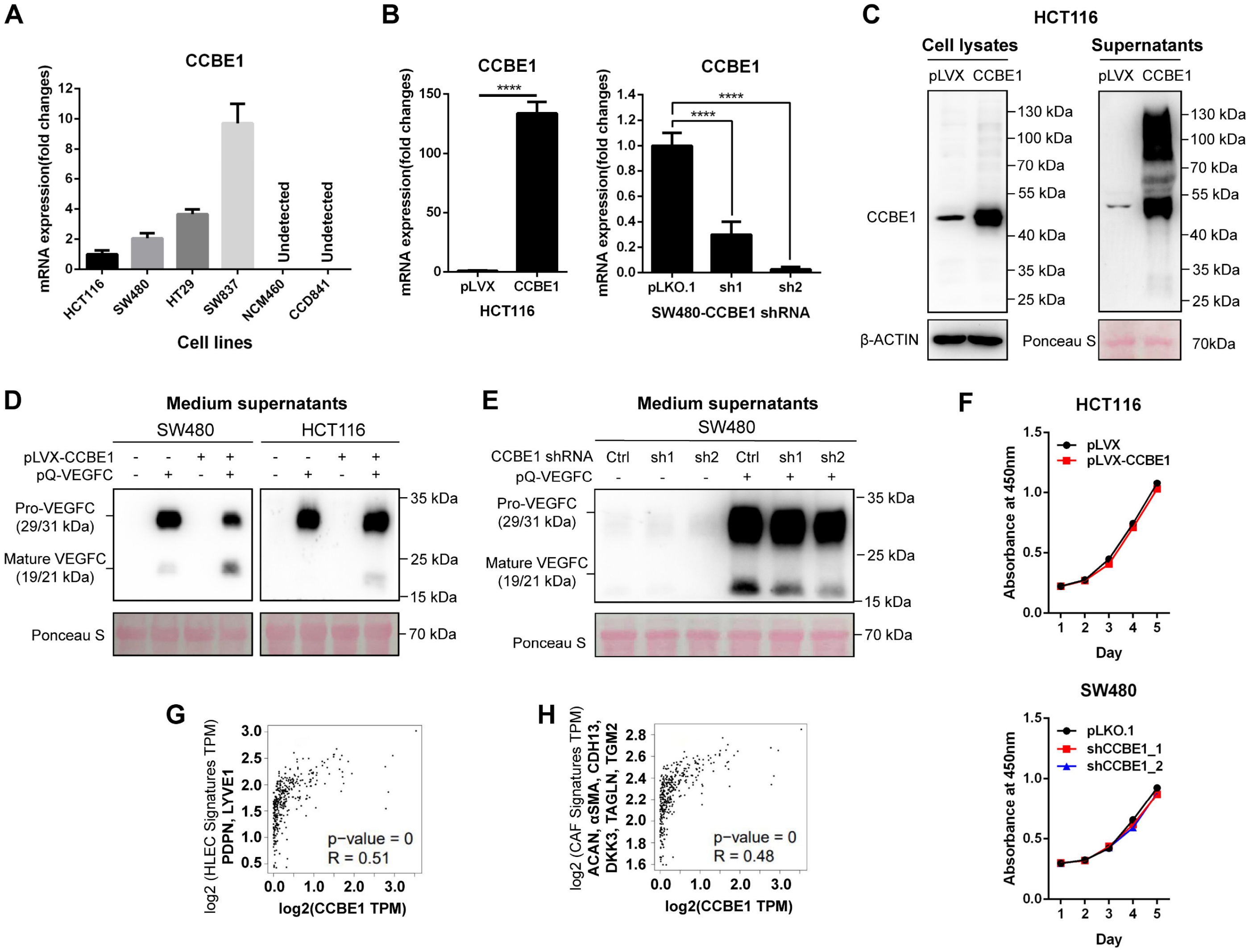

Figure S1. Related to Figure 1

(A) qPCR analysis of CCBE1 mRNA levels in four CRC cell lines (HCT116, SW480, HT29 and SW837) and two human normal colonic epithelial cell lines (NCM460 and CCD 841). (B) qPCR analysis of CCBE1 mRNA levels in the indicated stable HCT116 and SW480 cells. ****P<0.0001 by Student's t-test. (C) Western blot analysis of CCBE1 protein levels in cell lysates and supernatants of control and CCBE1-overexpressing HCT116 cells. (D, E) Western blot analysis of pro-VEGFC and mature VEGFC protein levels in supernatants from stable HCT116 and SW480 cells transduced with the indicated virus. CCBE1 overexpression in SW480 and HCT116 cells promotes the proteolysis of VEGFC, while CCBE1 knockdown in SW480 cells attenuates the proteolysis of VEGFC secreted by CRC cells. Ponceau S staining was used to control for equal loading of supernatant samples. (F) Cell counting kit-8 assays of stable HCT116 and SW480 cells transduced with the indicated virus. (G, H) Positive correlation between CCBE1 mRNA expression and the expression of HLEC markers (PDPN and LYVE1) and the expression of CAF markers (ACAN, αSMA, CDH13, DKK3, TAGLN, and TGM2) in the TCGA CRC dataset. Pearson's correlation was used to assess the statistical significance. Data were extracted from the GEPIA database.

Figure S2

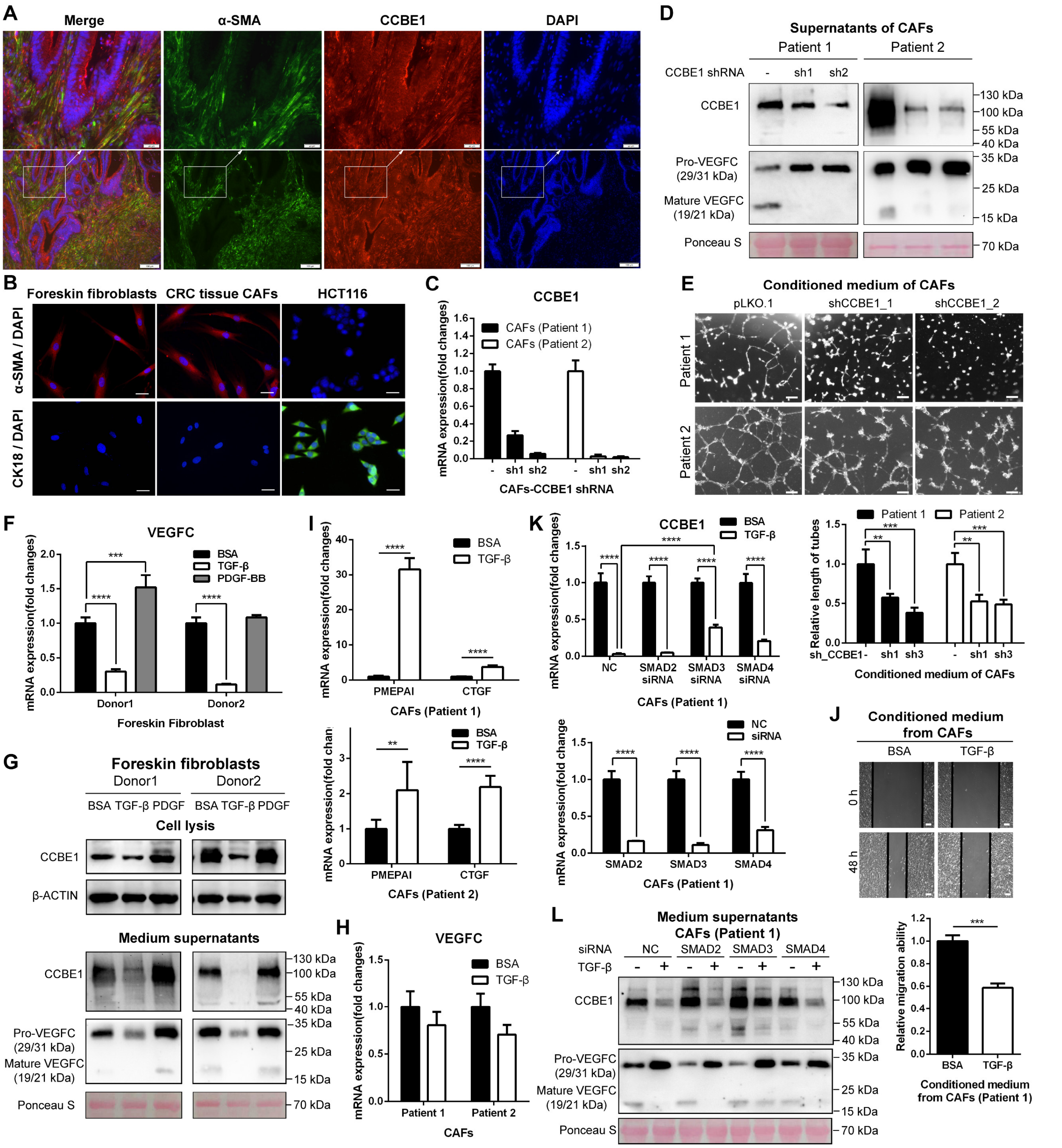

Figure S2. Related to Figure 3, 4 and 6.

(A) Immunofluorescent co-staining of CCBE1 and α-SMA in CRC tissue. Scale bars: 20 μm. (B) Immunofluorescence validation assay for isolated foreskin fibroblasts and CRC CAFs, with HCT116 cells as a control for epithelial cells. Scale bars: 20 μm. (C) qPCR analysis of CCBE1 mRNA levels in control and CCBE1-knockdown CAFs of two patients. (D) Western blot analysis of CCBE1, pro-VEGFC and mature VEGFC protein levels in supernatants from CCBE1-knockdown CAFs of two patients. (E) HLEC tube formation assay with conditioned medium from control and CCBE1-knockdown CAFs of two patients. Scale bars: 100 μm. **P<0.01, ***P<0.001 by Student's t-test. (F, G) Foreskin fibroblasts were treated with control BSA (0.1%), TGF-β (10 ng/ml) or PDGF-BB (20 ng/ml) for 72 h to generate CAFs in vitro. (F) qPCR analysis of VEGFC mRNA expression levels. ***P<0.001, ****P<0.0001 by Student's t-test. (G) Western blot analysis of CCBE1 protein in cell lysates and of

CCBE1, pro-VEGFC and mature VEGFC protein levels in supernatants from the indicated fibroblasts. Ponceau S staining was used as a loading control. (H) qPCR analysis of VEGFC expression in CAFs from two patients after treatment with control BSA (0.1%) or TGF-β (10 ng/ml) for 72 h. (I) qPCR analysis of PMEPAI and CTGF in CAFs from two patients after treatment with control BSA (0.1%) or TGF-β (10 ng/ml) for 72 h. **P<0.01, ****P<0.0001 by Student's t-test. (J) Would healing assay of HLECs cultured with conditioned medium of the indicated CAFs. The migration ability was calculated as the difference in blank area between 0 and 48 h. Scale bars: 20 μm. ***P<0.001 by Student's t-test. (K, L) CAFs were transfected with negative control (NC) or siRNA targeting SMAD2, SMAD3 or SMAD4. (K) qPCR analysis of SMAD2/3/4 and CCBE1 mRNA expression. (L) Western blot analysis of CCBE1, pro-VEGFC and mature VEGFC protein levels in supernatants from the indicated CAFs.

Figure S3

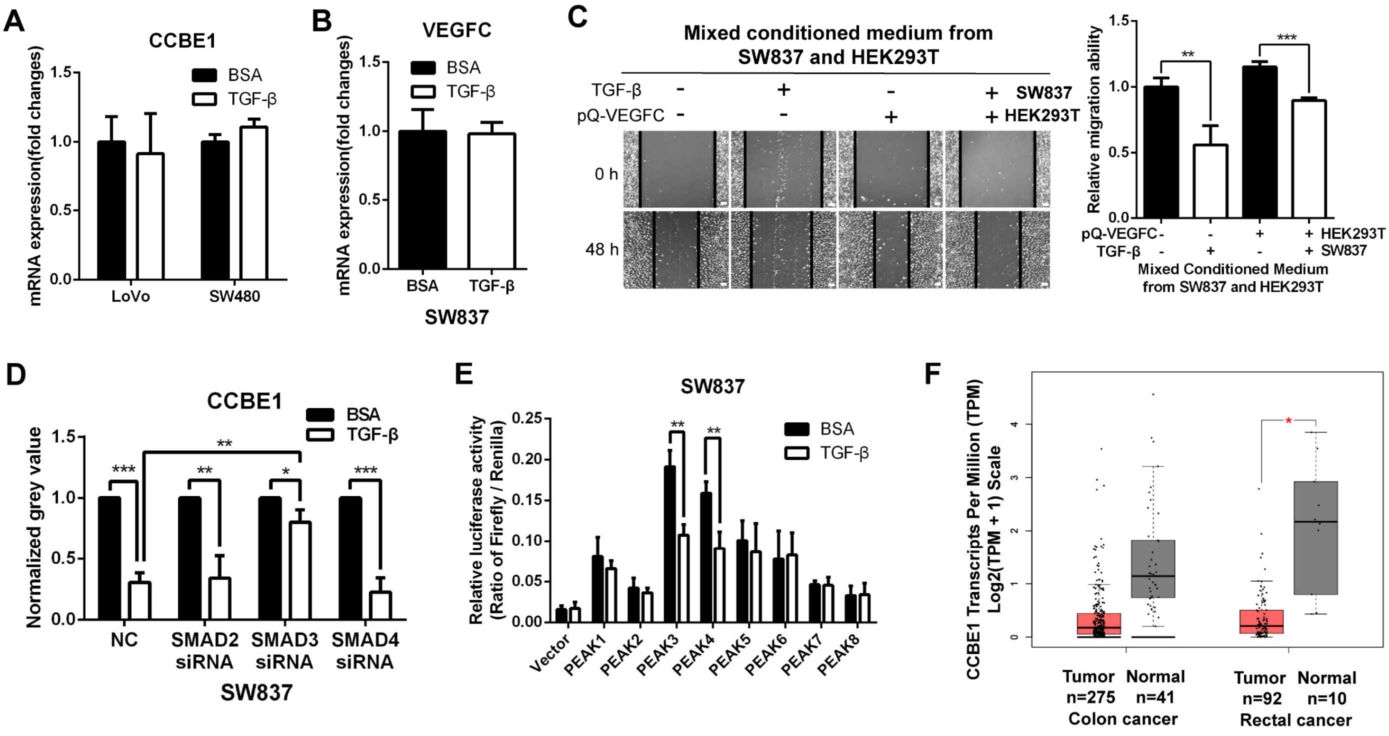

Figure S3. Related to Figure 3, 5 and 6.

(A) qPCR analysis of CCBE1 mRNA levels in LoVo and SW480 cells treated with control BSA (0.1%) or TGF-β (10 ng/ml) for 6 h. (B) qPCR analysis of VEGFC expression levels in SW837 cells treated with control BSA (0.1%) or TGF-β (10 ng/ml) for 6 h. (C) Wound healing assay of HLECs with the indicated conditioned medium. **P<0.01, ***P<0.001 by Student's t-test. Scale bars: 20 μm. (D) Statistical analysis of CCBE1 protein levels in the SW837 cells transfected with indicated siRNA after TGF-β treatment in three independent experiments by western blot analysis. The gray values of bands in the TGF-β groups were normalized to gray values of the BSA groups. *P<0.05, **P<0.01, ***P<0.001 by Student's t-test. (E) Relative luciferase activity of the SMAD3 potential binding peaks in CCBE1 gene locus-driven luciferase reporters in control BSA (0.1%) or TGF-β (10 ng/ml)-treated SW837 cells. **P<0.01 by Student's t-test. (F) Box-and-whisker plots of CCBE1 mRNA levels in normal colorectal mucosa and CRC tissues from the TCGA CRC dataset. Data were extracted from the GEPIA database.

Table S1

Table. S1 Correlation of CCBE1 expression in tumor cells with CRC patients' pathological and clinical features				
**Variables**	**CCBE1 expression in tumor cells**			**P-Values**
	All cases (n=277)	Low (n=112)	High (n=165)	
**Age (year)**				0.218[b]
≤68	140	54(38.6%)	86(61.4%)	
＞68	137	58(42.3%)	79(57.7%)	
**Gender**				0.446[b]
Male	153	66(43.1%)	87(56.9%)	
Female	124	46(37.1%)	78(62.9%)	
**Tumor site[a]**				**0.002[c]**
Proximal colon	66	16(24.2%)	50(75.8%)	
Distal colon	90	39(43.3%)	51(56.7%)	
Rectum	121	57(47.1%)	64(52.9%)	
**Pathology grade**				0.093[d]
Well differentiated	93	32(34.4%)	61(65.6%)	
Moderately differentiated	130	56(43.1%)	74(56.9%)	
Poorly differentiated	54	24(44.4%)	30(55.6%)	
**TNM staging**				
I	40	19(47.5%)	21(52.5%)	**0.025[d]**
II	112	56(50.0%)	56(50.0%)	
III	100	29(29.0%)	71(71.0%)	
IV	25	8(32.0%)	17(68.0%)	
Early stage(I/II)	152	75(49.3%)	77(50.7%)	**0.011[b]**
Late stage(III/IV)	125	37(29.6%)	88(70.4%)	
**Tumor infiltration depth**				0.809[b]
Limited under the serosa(T1/2/3)	147	60(40.8%)	87(59.2%)	
Penetrating the serosa(T4)	130	52(40.0%)	78(60.0%)	
**Regional lymph node metastasis**				**0.016[b]**
N0	161	78(48.4%)	83(51.6%)	
N1/N2	116	34(29.3%)	82(70.7%)	
**Distal metastasis**				0.615[b]
M0	252	104(41.3%)	148(58.7%)	
M1	25	8(32.0%)	17(68.0%)	
**CEA level[e]**				0.668[b]
0–10 ng/ml	203	84(41.4%)	119(58.6%)	
>10 ng/ml	68	25(36.8%)	43(63.2%)	

Abbreviations: TNM, tumor − node − metastasis; CEA, carcinoembryonic antigen. [a]Proximal colon tumors are those arising in the cecum, ascending colon, hepatic flexure or transverse colon; distal colon tumors are those arising in the splenic flexure, descending colon or sigmoid colon. [b]Mann-Whitney U Test. [c]Kruskal–Wallis. [d]Spearman. [e]Six patients did not have CEA level tested. The bold values indicate statistically significant (P<0.05).

Table S2

Table S2. Univariate and multivariate analysis of overall survival and disease-free survival (CCBE1 in tumor cells)								
Variables	OS				DFS			
	Univariate HR(95%CI)	P value	Multivariate HR(95%CI)	P value	Univariate HR(95%CI)	P value	Multivariate HR(95%CI)	P value
**Tumor infiltration depth**								
Limited under the serosa(T1/2/3)	1		1		1		1	
Penetrating the serosa(T4)	1.708(1.134-2.572)	0.01	1.327(0.862-2.042)	0.198	1.703(1.113-2.607)	0.014	1.371(0.876-2.146)	0.168
**Clinical stage**								
Early stage(I/II)	1		1		1			
Late stage(III/IV)	2.295(1.514-3.481)	<0.001	1.785(1.163-2.740)	**0.008**	2.263(1.468-3.489)	<0.001	1.818(1.164-2.840)	**0.009**
**Pathology grade**		0.028		0.051		0.034		0.057
Well differentiated	1		1		1		1	
Moderately differentiated	1.453(0.885-2.386)	0.139	1.365(0.824-2.263)	0.227	1.505(0.896-2.527)	0.122	1.483(0.874-2.513)	0.144
Poorly differentiated	2.146(1.225-3.759)	0.008	2.038(1.147-3.623)	**0.015**	2.174(1.211-3.904)	0.009	2.076(1.140-3.779)	**0.017**
**CEA level**								
0–10 ng/ml	1		1		1		1	
>10 ng/ml	2.693(1.775-4.085)	<0.001	2.309(1.494-3.567)	**<0.001**	2.453(1.572-3.825)	<0.001	2.138(1.350-3.386)	**0.001**
**CCBE1 expression in tumor cells**								
Low expression	1		1		1		1	
High expression	1.704(1.100-2.641)	0.017	1.729(1.093-2.735)	**0.019**	1.724(1.088-2.732)	0.02	1.704(1.051-2.762)	**0.031**

Table S3

Table S3. Univariate and multivariate analysis of overall survival and disease-free survival (CCBE1 in stroma)

Variables	OS				DFS			
	Univariate HR(95%CI)	P value	Multivariate HR(95%CI)	P value	Univariate HR(95%CI)	P value	Multivariate HR(95%CI)	P value
**Tumor infiltration depth**								
Limited under the serosa(T1/2/3)	1		1		1		1	
Penetrating the serosa(T4)	1.708(1.134-2.572)	0.01	1.332(0.863-2.056)	0.195	1.703(1.113-2.607)	0.014	1.368(0.872-2.145)	0.173
**Clinical stage**								
Early stage(I/II)	1		1		1			
Late stage(III/IV)	2.295(1.514-3.481)	<0.001	1.914(1.253-2.923)	**0.003**	2.263(1.468-3.489)	<0.001	1.930(1.243-2.997)	**0.003**
**Pathology grade**		0.028		0.08		0.034		0.081
Well differentiated	1		1		1		1	
Moderately differentiated	1.453(0.885-2.386)	0.139	1.310(0.793-2.163)	0.292	1.505(0.896-2.527)	0.122	1.409(0.835-2.377)	0.199
Poorly differentiated	2.146(1.225-3.759)	0.008	1.910(1.081-3.374)	**0.026**	2.174(1.211-3.904)	0.009	1.974(1.090-3.577)	**0.025**
**CEA level**								
0–10 ng/ml	1		1		1		1	
>10 ng/ml	2.693(1.775-4.085)	<0.001	2.292(1.477-3.557)	**<0.001**	2.453(1.572-3.825)	<0.001	2.120(1.333-3.372)	**0.002**
**CCBE1 expression in stroma**								
Low expression	1		1		1		1	
High expression	1.625(1.074-2.459)	0.021	1.640(1.077-2.499)	**0.021**	1.694(1.093-2.623)	0.018	1.631(1.046-2.543)	**0.031**

Table S4

Table S4. The sequence of siRNA, shRNA and primers used in this study

siSMAD2: GGAGUGCGCUUAUACUACA	ChIP primers	CCBE1 promoter primers
siSMAD3: GUCUACCAGUUGACCCGAA	PEAK1(F): CTACTTGGGTGACTGAGGCA	PEAK1(F): TTCTC TATCGATAGGTACC GTGGCTCATGCCTGTAATCC
siSMAD4: GGACAUUCAAUUCAAACCA	PEAK1(R): GCTGGAGTGTAGTGGTGGAA	PEAK1(R): GCAGATCGCAGATCTCGAG ACCTCCCAATCAAATGGTCA
shCCBE1-1; GTTCCCTTTACCTCAGGAATT	PEAK2(F): CTTGGGAAGGAATGCTCAGC	PEAK2(F): TTCTC TATCGATAGGTACC GGCATGGAAGACAACCCTGT
shCCBE1-2: GAGGGAGTGAATGATTGATTT	PEAK2(R): GGGAATGGGTGTCAAGGGTA	PEAK2(R): GCAGATCGCAGATCTCGAG CCCCATGTAATAAAAAGTAACACTGA
CCBE1(F): 5'-AAGTCTTCAGGCGAGCTCACC-3'	PEAK3(F): ATCTACACACCCAGACAGCC	PEAK3(F): TTCTC TATCGATAGGTACC TTGTTAAGTCTTTTATCTTAGGGCATC
CCBE1(R): 5'- GTTGTCCGTGCACTGCTGTTC-3'	PEAK3(R): GTCACTGCCACACCCAATTC	PEAK3(R): GCAGATCGCAGATCTCGAG ATCACACCACTGCACTCCAA
VEGFC(F): 5'-CAGCACGAGCTACCTCAGCAAG-3'	PEAK4(F): TCTGGCTCTAGTAACGGCTG	PEAK4(F): TTCTC TATCGATAGGTACC TGCTGGAATTACAGGCATGA
VEGFC(R): 5'-TTTAGACATGCATCGGCAGGAA-3'	PEAK4(R): TGCAGAATTCCAGGCTACCA	PEAK4(R): GCAGATCGCAGATCTCGAG GGCTACTTTCCCTACAGAAACC
PMEPAI(F): 5'- TGTCAGGCAACGGAATCCC-3'	PEAK5(F): TGTGAAGAAGAGAGCAGGGG	PEAK5(F): TCTC TATCGATAGGTACCGTTGTGGTGAGCCAAGATCA
PMEPAI(R): 5'-CAGGTACGGATAGGTGGGC-3'	PEAK5(R): TGCTCTCAGAGGGTTGACAG	PEAK5(R): GCAGATCGCAGATCTCGAG GCAGCTTCTCAGAGGTCCAG
CTGF(F): 5'-AGGAGTGGGTGTGTGACGA-3'	PEAK6(F): GGGCTTAGCTCTCATGGTCT	PEAK6(F): TCTC TATCGATAGGTACC ACTCATCCTCCTGCCTCTCA
CTGF(R): 5'-CCAGGCAGTTGGCTCTAATC-3'	PEAK6(R): AGGTGACCAGAACAGACAGG	PEAK6(R): GCAGATCGCAGATCTCGAG GCACTTTCTCACAAGGCACA
SMAD2(F): 5'- CGTCCATCTTGCCATTCACG-3'	PEAK7(F): GCTGTGTTTCCCCAATGACT	PEAK7(F): TCTC TATCGATAGGTACCTGCTTGGATTACAGGCATGA
SMAD2(R): 5'-CTCAAGCTCATCTAATCGTCCTG-3'	PEAK7(R): GGGGTGAGAACAAGTCAATCC	PEAK7(R): GCAGATCGCAGATCTCGAG CTGACTGTTCTGATGCCAGAC
SMAD3(F): 5'-TGGACGCAGGTTCTCCAAAC-3'	PEAK8(F): ATTGCTGGCAGTGGGAAAAG	PEAK8(F): TCTC TATCGATAGGTACC TCTGAGCTCTGCTTTGTCCA
SMAD3(R): 5'-CCGGCTCGCAGTAGGTAAC-3'	PEAK8(R): GCCCAGTGACACGTAGCTAT	PEAK8(R): GCAGATCGCAGATCTCGAG TTTGGTCGCACTTACAGCAA
SMAD4(F): 5'-CTCATGTGATCTATGCCCGTC-3'		
SMAD4(R): 5'-AGGTGATACAACTCGTTCGTAGT-3'		
